# Theranostic Small-Molecule Prodrug Conjugates for Targeted Delivery and Controlled Release of Toll-like Receptor 7 Agonists

**DOI:** 10.3390/ijms23137160

**Published:** 2022-06-28

**Authors:** Sashi Debnath, Guiyang Hao, Bing Guan, Pawan Thapa, Justin Hao, Hans Hammers, Xiankai Sun

**Affiliations:** 1 Department of Radiology, University of Texas Southwestern Medical Center, Dallas, TX 75390, USA; sashi.debnath@utsouthwestern.edu (S.D.); guiyang.hao@utsouthwestern.edu (G.H.); guanbing27@163.com (B.G.); pawan.thapa@utsouthwestern.edu (P.T.); hao@my.unt.edu (J.H.); 2 Texas Academy of Mathematics and Science, University of North Texas, Denton, TX 76203, USA; 3 Department of Internal Medicine, University of Texas Southwestern Medical Center, Dallas, TX 75390, USA; 4 Kidney Cancer Program, University of Texas Southwestern Medical Center, Dallas, TX 75390, USA; 5 Advanced Imaging Research Center, University of Texas Southwestern Medical Center, Dallas, TX 75390, USA

**Keywords:** toll-like receptors, agonist, controlled release, prodrug, theranostic, immunomodulatory, legumain cleavable linker, positron emission tomography, prostate cancer

## Abstract

We previously reported the design and synthesis of a small-molecule drug conjugate (SMDC) platform that demonstrated several advantages over antibody–drug conjugates (ADCs) in terms of in vivo pharmacokinetics, solid tumor penetration, definitive chemical structure, and adaptability for modular synthesis. Constructed on a tri-modal SMDC platform derived from 1,3,5-triazine (TZ) that consists of a targeting moiety (Lys-Urea-Glu) for prostate-specific membrane antigen (PSMA), here we report a novel class of chemically identical theranostic small-molecule prodrug conjugates (T-SMPDCs), [^18/19^F]F-TZ(PSMA)-LEGU-TLR7, for PSMA-targeted delivery and controlled release of toll-like receptor 7 (TLR7) agonists to elicit de novo immune response for cancer immunotherapy. In vitro competitive binding assay of [^19^F]F-TZ(PSMA)-LEGU-TLR7 showed that the chemical modification of Lys-Urea-Glu did not compromise its binding affinity to PSMA. Receptor-mediated cell internalization upon the PSMA binding of [^18^F]F-TZ(PSMA)-LEGU-TLR7 showed a time-dependent increase, indicative of targeted intracellular delivery of the theranostic prodrug conjugate. The designed controlled release of gardiquimod, a TLR7 agonist, was realized by a legumain cleavable linker. We further performed an in vivo PET/CT imaging study that showed significantly higher uptake of [^18^F]F-TZ(PSMA)-LEGU-TLR7 in PSMA^+^ PC3-PIP tumors (1.9 ± 0.4% ID/g) than in PSMA^−^ PC3-Flu tumors (0.8 ± 0.3% ID/g) at 1 h post-injection. In addition, the conjugate showed a one-compartment kinetic profile and in vivo stability. Taken together, our proof-of-concept biological evaluation demonstrated the potential of our T-SMPDCs for cancer immunomodulatory therapies.

## 1. Introduction

Toll-like receptors (TLRs) are a class of evolutionarily conserved pattern-recognition receptors that play a critical bridging role in the innate and adaptive immunity to combat a variety of illnesses resulting from the infection of bacteria, viruses, and other microbes [1,2]. Of the 10 human and 13 mice TLRs that have been identified, some are located on the cell membrane (e.g., TLR1, TLR2, TLR4, TLR5, TLR6, and TLR10), while others (e.g., TLR3, TLR7, TLR8, and TLR9) are present inside the endosomes, where they recognize nucleic acids and nucleotides of intracellular pathogens [3,4]. Elicited by pathogen-associated molecular patterns (PAMPs), xenobiotic-associated molecular patterns (XAMPs), and danger-associated molecular patterns (DAMPs), TLR pathway activation results in the release of pro-inflammatory chemokines, cytokines (e.g., TNF-α, IL-1β, IL-6, IL-12), and type I interferon (IFN) as the host’s anti-microbial or anti-tumor responses [5]. Additionally, TLRs connect the innate and adaptive immune response by the up-regulation of co-stimulatory signals on antigen-presenting dendritic cells (DCs) to intensify both T- and B-cell immune responses [6]. Given that the TLR signaling irregularity leads to the progression of many diseases (e.g., autoimmune diseases, infection, sepsis, chronic inflammation, and cancer) [7,8,9,10] and that upregulation of TLRs has identified in many cancer types (e.g., colon cancer, hepatocellular carcinoma, breast cancer, and prostate cancer) [11,12,13,14,15], TLR pathway activation has been leveraged as a promising strategy for the development of targeted therapeutics [2,16].

**Scheme 1:**
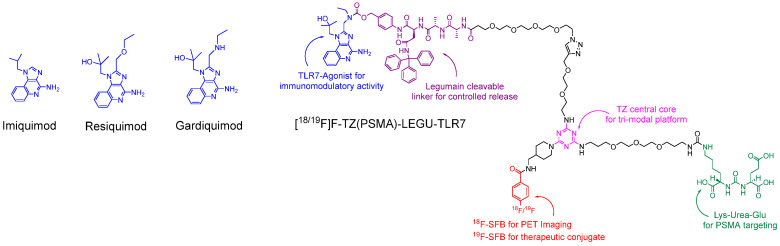
Structures of imidazoquinoline-derived TLR7 agonists and the chemically identical T-SMPDCs presented in this work, [^18/19^F]F-TZ(PSMA)-LEGU-TLR7.

Among the TLRs, TLR7 has served as an attractive and productive druggable target for synthetic TLR agonist development. To date, a large number of TLR7 agonists have been reported [17,18]. Primarily expressed in the cells of the innate immune system that are virtually present in all solid tumors, TLR7 is an intracellular target. As such, one of the essential criteria for TLR7-targeted drug design is that the drug payload must be able to cross the cellular membrane to activate TLR7, which in tandem will interact with myeloid differentiation protein 88 (MyD88) and translocate the NF-κB transcription factor into the nucleus to release pro-inflammatory chemokines and cytokines [19]. Most clinically tested TLR7 targeting small-molecule agonists are imidazoquinoline derivatives (Scheme 1), among which imiquimod is the first Food and Drug Administration (FDA)-approved drug (in 1997) to treat basal cell carcinoma and genital warts [20,21]. Followed by imiquimod’s approval, many other potent imidazoquinoline derivatives, such as resiquimod and gardiquimod, have been reported with improved potency and solubility [21,22].

However, while the highly potent imidazoquinoline agents can be potentially used as immunomodulatory agents and vaccine adjuvants for infectious diseases, the dose limiting toxicities and general inflammatory responses of the host observed in their clinical trials are so severe that the systemic administration (i.e., untargeted infusion into the vein) has been abandoned [23,24,25,26,27]. In addition, the agents exhibited poor pharmacokinetic characteristics mainly due to their poor solubility [28]. Consequently, to reach an effective dose for anti-tumor treatment, current strategies require the agents to be directly injected into tumors multiple times on a weekly basis. Given the logistical barriers, risks of bleeding/organ damage/infection, this practice significantly hampers the clinical application of these agents. In addition, the intra-tumoral injection is not applicable to deep-seated metastases, and the fact that tumors are inherently heterogeneous makes the approach of little clinical value.

**Scheme 2:**
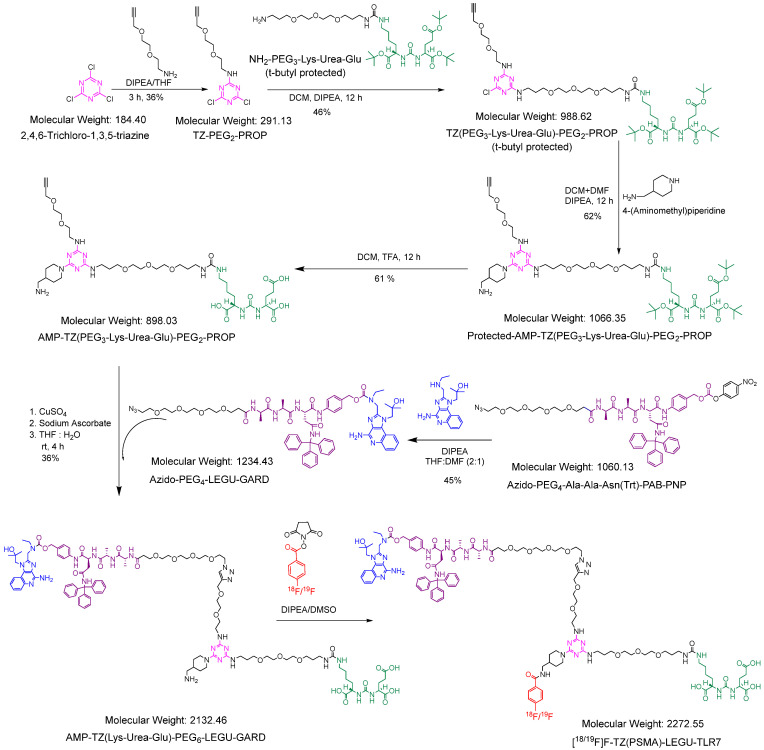
Synthetic routes to the key intermediate AMP-TZ(Lys-Urea-Glu)-PEG_6_-LEGU-GARD and chemically identical theranostic pair of [^18/19^F]F-TZ(PSMA)-LEGU-TLR7. DIPEA: N,N-diisopropylethylamine; THF: tetrahydrofuran; TZ: 2,4,6-trichloro-1,3,5-triazine; PEG: polyethylene glycol; PROP: propargyl; Lys: lysine; Glu: glutamic acid; DCM: dichloromethane; DMF: N,N-dimethylformamide, AMP: 4-aminomethyl piperidine; TFA: trifluoroacetic acid; rt: room temperature, PAB: para aminobenzoic acid; PNP: para nitrophenol; Ala: alanine; Asn: asparagine; Trt: triphenyl methyl; LEGU: legumain; GARD: gardiquimod; DMSO: dimethyl sulfoxide.

We reason that targeted systemic delivery of the TLR7 agonists via our previously reported small-molecule drug conjugate (SMDC) platform [29] could surmount the roadblock that impedes their clinical application. In this work, we present a uniquely designed chemically identical pair of theranostic small-molecule prodrug conjugates (T-SMPDCs), [^18/19^F]FB-AMP-TZ(PEG_3_-Lys-Urea-Glu)-PEG_6_-LEGU-GARD ([^18/19^F]F-TZ(PSMA)-LEGU-TLR7), constructed on a tri-modal molecule, 2,4,6-trichloro-1,3,5-triazine (TZ), where the halogen functionality was leveraged for modular synthesis of the conjugation (Scheme 2). The water-soluble short polyethylene glycol (PEG) linkers were employed to optimize the in vivo kinetics of the conjugate as necessary [30]. Urea-based Lys-Urea-Glu [31], which is a commonly used targeting moiety for prostate-specific membrane antigen (PSMA), serves as a model vector for cancer-targeted delivery of TLR7 agonists. Of note, the well-observed PSMA-mediated internalization mechanism upon ligand-binding [32] is leveraged for the intracellular delivery of the TLR7 payload carried by the T-SMPDCs. To enable the designed controlled release [33,34,35], we incorporate a legumain-cleavable linker, Azido-PEG_4_-Ala-Ala-Asn(Trt)-PAB-PNP (Azido-PEG_4_-LEGU), which is stable in the blood, as reported in many antibody drug conjugates (ADCs), to minimize the off-target toxicities [36,37], but undergoes a traceless release (self-immolation) of drugs upon interacting with legumain, a lysosomal endopeptidase. For the proof-of-concept, in this work we chose gardiquimod (GARD) as a model TLR7 agonist to construct our prodrug conjugates, which was coupled to the legumain-cleavable linker. At the last step, an ^18/19^F-labeled 4-fluorobenzoate ([^18/19^F]SFB) [38,39] was incorporated into one of the TZ’s arm to complete the modular synthesis of a pair of chemically identical T-SMPDCs, [^18/19^F]F-TZ(PSMA)-LEGU-TLR7. The T-SMPDC pair with identical in vivo properties are designed for targeted theranostic application. While the radioactive conjugate, [^18^F]F-TZ(PSMA)-LEGU-TLR7), is intended for positron emission tomography (PET) imaging, its cold counterpart, [^19^F]F-TZ(PSMA)-LEGU-TLR7, can be formulated for cancer immunomodulatory therapy. Herein, we present our modular synthesis and proof-of-concept evaluations of [^18/19^F]F-TZ(PSMA)-LEGU-TLR7 for potential theranostic applications in prostate cancer.

## 2. Results

### 2.1. Structural Design and Synthesis

To enable the modular synthesis of T-SMPDCs and avoid potential steric hindrance that might result from the assembly of three designed functionalities, we introduced three different spacers between the tri-modal TZ core and each functional moiety (Scheme 2, Schemes S1–S3, and Figures S1–S21). In brief, propargyl-PEG_2_-amine was conjugated with TZ in (1:1) stoichiometric fashion to afford monosubstituted TZ-PEG_2_-PROP, in which the propargyl group (PROP) was introduced for azido-based click chemistry. Then TZ-PEG_2_-PROP was coupled with NH_2_-PEG_3_-Lys-Urea-Glu (t-butyl protected) by replacing a second chloride to produce TZ(PEG_3_-Lys-Urea-Glu)-PEG_2_-PROP (t-butyl protected) in 46% yield, followed by reaction with 4-(aminomethyl)piperidine (AMP) to afford protected AMP-TZ(PEG_3_-Lys-Urea-Glu)-PEG_2_-PROP in 62% yield. Finally, deprotection of protected AMP-TZ(PEG_3_-Lys-Urea-Glu)-PEG_2_-PROP with trifluoroacetic acid (TFA) produces the PSMA-targeting small-molecule conjugate platform, AMP-TZ(PEG_3_-Lys-Urea-Glu)-PEG_2_-PROP in 61% yield, which bears PROP and AMP moieties for further modular synthesis towards T-SMPDCs.

#### 2.1.1. Synthesis of the Key Intermediate AMP-TZ(Lys-Urea-Glu)-PEG_6_-LEGU-GARD

As shown in Scheme 2, the reaction of legumain-cleavable linker azido-PEG_4_-LEGU with GARD in amine media produced azido-PEG_4_-LEGU-GARD in 45% yield. Under a typical click-reaction condition, AMP-TZ(PEG_3_-Lys-Urea-Glu)-PEG_2_-PROP was conjugated with Azido-PEG_4_-LEGU-GARD to produce the key intermediate AMP-TZ(Lys-Urea-Glu)-PEG_6_-LEGU-GARD in 36% yield.

#### 2.1.2. Synthesis of [^19^F]F-TZ(PSMA)-LEGU-TLR7

Although AMP-TZ(Lys-Urea-Glu)-PEG_6_-LEGU-GARD has two primary amines (aromatic and aliphatic) available for new amide bond formation, the aliphatic amine next to the piperidine ring is naturally more reactive [40]. The final prodrug conjugate of T-SMPDC, [^19^F]F-TZ(PSMA)-LEGU-TLR7, was synthesized by reacting AMP-TZ(Lys-Urea-Glu)-PEG_6_-LEGU-GARD with [^19^F]SFB in 57% yield (Scheme 2).

### 2.2. Radiochemistry

The multistep radiosynthesis of [^18^F]T-SMPDC was initiated with ~55.5 GBq of [^18^F]fluoride which was helpful for reproducible radiochemical yield and molar activity. The synthetic strategy developed for [^19^F]F-TZ(PSMA)-LEGU-TLR7 was readily adapted to couple [^18^F]SFB with AMP-TZ(Lys-Urea-Glu)-PEG_6_-LEGU-GARD to afford [^18^F]F-TZ(PSMA)-LEGU-TLR7. The production of [^18^F]SFB was optimized in an automated Synthra RNplus synthesis module (Synthra GmbH, Hamburg, Germany). The radiosynthesis of [^18^F]F-TZ(PSMA)-LEGU-TLR7 was completed within 90 min from the end of [^18^F]SFB production. After purification, [^18^F]F-TZ(PSMA)-LEGU-TLR7 was formulated with phosphate-buffered saline (PBS) containing 10% ethanol (EtOH) (EtOH/PBS: 10/90 *v*/*v*) for subsequent in vitro and in vivo studies.

#### 2.2.1. Automated Radiosynthesis of [^18^F]SFB

The synthesis of [^18^F]SFB was accomplished by a three-step one-pot method previously reported in a synthra RNplus synthesis module (Scheme S2) [39]. In brief, 4-(ethoxycarboxyl)-N,N,N-trimethylbenzenaminium trifluoromethanesulfonate was radiofluorinated to produce ethyl 4-[^18^F]fluorobenzoate and then the ester group was hydrolyzed by tetrapropylammonium hydroxide (TPAH) to afford 4-[^18^F]fluorobenzoic acid. The free carboxylic acid was then activated with O-(N-succinimidyl)-1,1,3,3-tetramethyluronium tetrafluoroborate (TSTU) to produce [^18^F]SFB. HPLC purified [^18^F]SFB was completely evaporated and reconstituted to improved radiochemical yield in next radiolabeling reaction.

#### 2.2.2. Radiosynthesis of [^18^F]F-TZ(PSMA)-LEGU-TLR7

Radiolabeling of AMP-TZ(Lys-Urea-Glu)-PEG_6_-LEGU-GARD with [^18^F]SFB was performed at 55 °C in two different solvents, dimethylformamide (DMF) or dimethyl sulfoxide (DMSO). In DMSO, we were able to obtain [^18^F]F-TZ(PSMA)-LEGU-TLR7 (Scheme 2) in higher radiochemical yields (RCY: DMSO, 37% vs. DMF, 26%; *n* > 3). The chemical identity of [^18^F]F-TZ(PSMA)-LEGU-TLR7 was confirmed by radio-HPLC with retention time at 17.5 min, which had been calibrated with the HPLC retention time (17 min) of [^19^F]F-TZ(PSMA)-LEGU-TLR7 measured by UV at 254 nm (Figure S22). Of note, the UV and radioactivity detectors were in a series connection at ~0.5 min apart under the HPLC conditions. The radiochemical purity of [^18^F]F-TZ(PSMA)-LEGU-TLR7 was > 99% as determined by radio-HPLC and radio-TLC. Measured log*P*_oct/PBS_ value was 0.48. The calculated molar activity of [^18^F]F-TZ(PSMA)-LEGU-TLR7 was 15.2 ± 0.5 GBq/μmol (*n* = 3) at the end of synthesis.

### 2.3. In Vitro Assays

#### 2.3.1. Human Serum Treatment Showed No Decomposition of [^18^F]F-TZ(PSMA)-LEGU-TLR7

To test the in vitro stability of [^18^F]F-TZ(PSMA)-LEGU-TLR7 in serum, 1.85 MBq of the radioactive conjugate was incubated with 400 μL of human serum at 37 °C. The mixture was sampled at 1, 2, and 4 h (*n* = 3) for radio-HPLC assay. No decomposition of [^18^F]F-TZ(PSMA)-LEGU-TLR7 was observed out to 4 h in human serum.

#### 2.3.2. Competitive Cell-Binding Assay Demonstrated the PSMA Binding Affinity of [^19^F]F-TZ(PSMA)-LEGU-TLR7 Was Not Compromised

**Figure 1:**
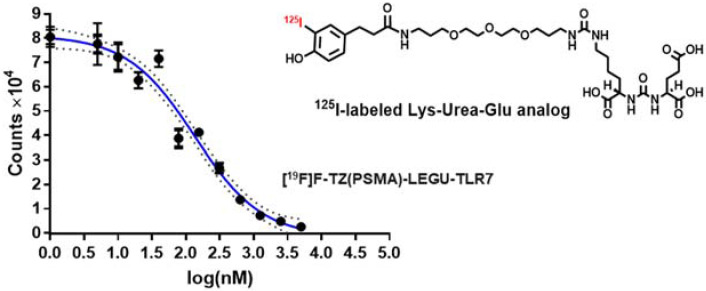
Competitive PSMA binding affinity measurement of [^19^F]F-TZ(PSMA)-LEGU-TLR7 using PSMA^+^ PC3-PIP cells and competitive radioligand ^125^I-labeled Lys-Urea-Glu. The IC_50_ values was measured to be 134 ± 37 nM by nonlinear regression of the data fitting using GraphPad Prism 7.04 (coefficient of determination R^2^ = 0.97). Data presented as counts ± s.d. (*n* = 3).

A competitive cell-binding assay was performed to measure the binding affinity of [^19^F]F-TZ(PSMA)-LEGU-TLR7 towards PSMA using PSMA^+^ PC3-PIP cells and ^125^I-labeled Lys-Urea-Glu as the competitive radioligand. PC3-PIP cells were incubated with ^125^I-labeled Lys-Urea-Glu in 96 MultiScreen-DV filter plate, and [^19^F]F-TZ(PSMA)-LEGU-TLR7 was added in a series of dilutions. The IC_50_ value was measured by the concentration of [^19^F]F-TZ(PSMA)-LEGU-TLR7 required to displace 50% of PSMA^+^ cell bound ^125^I-labeled Lys-Urea-Glu. As shown in Figure 1, [^19^F]F-TZ(PSMA)-LEGU-TLR7 inhibits the PSMA binding of ^125^I-labeled Lys-Urea-Glu in a concentration dependent manner. The calculated IC_50_ value of [^19^F]F-TZ(PSMA)-LEGU-TLR7 was 134 ± 37 nM, which is close to that of our previously reported T-SMDC, ^nat^Ga-NO3A-DM1-Lys-Urea-Glu (187 ± 41 nM) [29], confirming that the PSMA targeting property of Lys-Urea-Glu was not compromised after presented onto the T-SMPDC platform.

#### 2.3.3. [18.F]F-TZ(PSMA)-LEGU-TLR7 Showed PSMA Specific Cell Uptake and PSMA-Mediated Internalization

**Figure 2:**
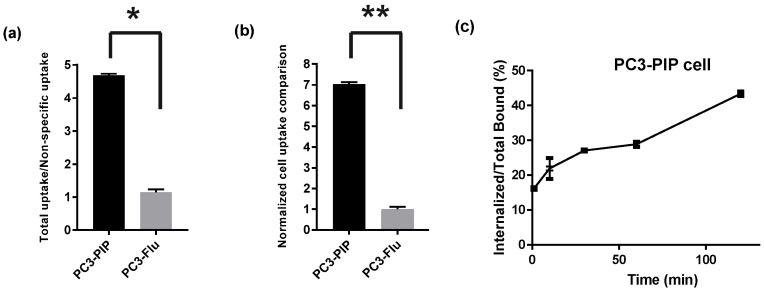
In vitro cell assays of [^18^F]F-TZ(PSMA)-LEGU-TLR7. (**a**) Total cell uptake vs. nonspecific uptake of [^18^F]F-TZ(PSMA)-LEGU-TLR7 in the absence and presence of Lys-Urea-Glu (1 mM). (**b**) Normalized PSMA specific uptake (no blocking) of [^18^F]F-TZ(PSMA)-LEGU-TLR7 (the uptake change in PC-3-Flu cells was set at 1.0). (**c**) Internalization of [^18^F]F-TZ(PSMA)-LEGU-TLR7 in PSMA^+^ PC3-PIP cells. Data presented as average counts ± s.d. (*n* = 3). * *p* = 0.0004, ** *p* = 0.0004 (unpaired t-tests performed by GraphPad Prizm 7.04).

The PSMA-specific uptake of [^18^F]F-TZ(PSMA)-LEGU-TLR7 was determined by two methods. As shown in Figure 2a, the PSMA-specific uptake of [^18^F]F-TZ(PSMA)-LEGU-TLR7 was measured in PSMA^+^ PC3-PIP vs. PSMA^−^ PC3-Flu. The specific vs. nonspecific uptake ratio of [^18^F]F-TZ(PSMA)-LEGU-TLR7 was ~4.5. The other method used the PSMA-binding blockade of 1 mM of Lys-Urea-Glu, resulting in a 7-fold reduction of PSMA-binding. In other words, the PSMA specific uptake of [^18^F]F-TZ(PSMA)-LEGU-TLR7 was ~7 times higher in PC3-PIP than PC3-Flu cells (Figure 2b). Furthermore, we observed a time-dependent PSMA-mediated internalization of [^18^F]F-TZ(PSMA)-LEGU-TLR7 (Figure 2c), demonstrating that the drug payload can be intracellularly delivered to reach the action target by our T-SMPDC platform.

#### 2.3.4. Legumain-Enzyme-Induced GARD Release from [^19^F]F-TZ(PSMA)-LEGU-TLR7

To test if the T-SMPDC platform is capable of controlled release of GARD upon legumain-catalyzed cleavage, we performed an in vitro GARD release assay using a previously published method [41]. [^19^F]F-TZ(PSMA)-LEGU-TLR7 (150 μg) was dissolved in 15 μL of DMF and diluted to 400 μL with the cleavage buffer (0.1 M citrate pH 5.5) followed by the addition of 300 μg of cysteine. Murine legumain (7 μg in 25 mM tris buffer) was added to the mixture. After 1 h incubation at 37 °C, the sample showed a quasi-molecular ion peak of 365.07 [M + 2H_2_O]^+^, the molecular mass of hydrated GARD (Figure S23) released from [^19^F]F-TZ(PSMA)-LEGU-TLR7 [42]. The legumain-controlled release of GARD was further confirmed by the absence of the molecular ion peak at 365.07 when [^19^F]F-TZ(PSMA)-LEGU-TLR7 was incubated with the buffer only under the same condition (Figure S24).

### 2.4. In Vivo Evaluation of [^18^F]F-TZ(PSMA)-LEGU-TLR7

The in vivo studies were performed with [^18^F]F-TZ(PSMA)-LEGU-TLR7 to assess the in vivo kinetics, stability, metabolism, biodistribution, and tumor-targeting properties of the T-SMPDCs.

#### 2.4.1. [^18^F]F-TZ(PSMA)-LEGU-TLR7 Showed Reasonable In Vivo Stability and Was Mainly Excreted from Kidneys

**Figure 3:**
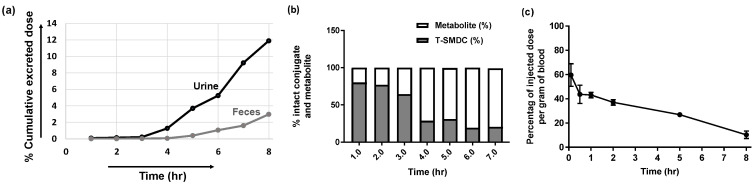
(**a**) Time-dependent clearance of [^18^F]F-TZ(PSMA)-LEGU-TLR7 from the body measured by cumulative radioactivity found in the urine and feces in terms of percentage of the injected dose (%ID) (*n* = 5). (**b**) Metabolites and intact [^18^F]F-TZ(PSMA)-LEGU-TLR7 found in the urine. Data presented as area under the curve in radio TLC. (**c**) Time-activity-curve (TAC) in the blood after intravenous injection of [^18^F]F-TZ(PSMA)-LEGU-TLR7 into mice (*n* = 3 in each time point); data presented as average %ID/g ± s.d. (*n* = 3).

The clearance and urine metabolite assays were performed in BALB/c mice (*n* = 5) after an intravenous injection of ~ 7.4 MBq of [^18^F]F-TZ(PSMA)-LEGU-TLR7 into each mouse. The mice were housed in a metabolic cage, from which urine and feces were collected for radio-TLC and gamma counting at 1, 2, 3, 4, 5, 6, 7, and 8 h post-injection (p.i). At 8 hr p.i., ~12% and ~3% of the injected dose (ID) were found in urine and feces, respectively (Figure 3a), indicating a relatively slow clearance profile of [^18^F]F-TZ(PSMA)-LEGU-TLR7, which could be beneficial to the therapeutic purpose.

We also analyzed the proportions of intact [^18^F]F-TZ(PSMA)-LEGU-TLR7 and its in vivo metabolites in the urine collected at each individual time point. As shown in Figure 3b, out to 3 hr p.i., ~70% of the activity excreted in the urine was found to be intact [^18^F]F-TZ(PSMA)-LEGU-TLR7, indicating its reasonable in vivo stability. In addition, we observed an increasing proportion of metabolites in the urine over the time course. Not surprisingly, the metabolites were all more hydrophilic than [^18^F]F-TZ(PSMA)-LEGU-TLR7.

#### 2.4.2. In Vivo Tissue Distribution Kinetics of [^18^F]F-TZ(PSMA)-LEGU-TLR7 Showed a One-Compartment Profile with Blood Circulation Half-Life of 8.2 h

The kinetic behavior was assessed after the intravenous injection of ~ 1.85 MBq of [^18^F]F-TZ(PSMA)-LEGU-TLR7 in each BALB/c mouse. Blood samples were collected from the submandibular vein at 0.1, 0.5, 1, 2, 5, and 8 h p.i. (*n* = 3 in each time point) to measure the activity in the blood by gamma counter. The time-activity-curve (TAC) in the blood (Figure 3c) shows a typical one-compartment in vivo kinetic profile with the blood circulation half-life (t_1/2_) estimated to be 8.2 h. The in vivo kinetic behavior is consistent with the clearance profile (Figure 3a), likely due to the structural features of [^18^F]F-TZ(PSMA)-LEGU-TLR7. 

#### 2.4.3. [^18^F]F-TZ(PSMA)-LEGU-TLR7 Demonstrated PSMA-Specific Uptake and Retention in PSMA^+^ Tumors

**Figure 4:**
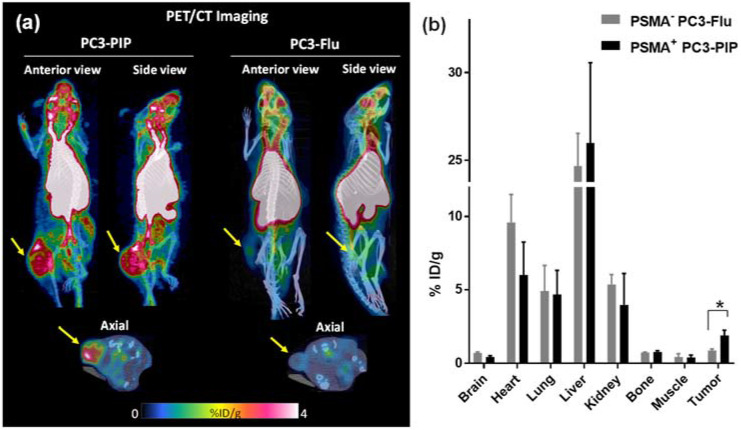
(**a**) Representative PET/CT images of SCID mice bearing PSMA^+^ PC3-PIP and PSMA^−^ PC3-Flu xenografts at 1 h p.i. of [^18^F]F-TZ(PSMA)-LEGU-TLR7. Yellow arrows indicate the tumor location. (**b**) Quantitative analysis of tissues of interest. Data presented as average %ID/g ± s.d. (*n* = 3). * *p* = 0.02 (unpaired t-tests performed by GraphPad Prizm 7.04).

The in vivo PET/CT imaging evaluation of [^18^F]F-TZ(PSMA)-LEGU-TLR7 was performed in severe combined immunodeficiency (SCID) mice subcutaneously bearing PSMA^+^ PC3-PIP and PSMA^−^ PC3-Flu xenografts. At 1 h p.i. of [^18^F]F-TZ(PSMA)-LEGU-TLR7, the PET imaging data was acquired for 15 min followed by a CT scan to obtain the anatomical information. Shown in Figure 4a are representative PET/CT images (1 h p.i.) presented in the format of maximum intensity projections (MIP). Clearly, the PSMA^+^ PC3-PIP tumors were visualized by PET imaging with [^18^F]F-TZ(PSMA)-LEGU-TLR7 but not the PSMA^−^ ones, indicative of the expected uptake specificity. Indeed, quantitative imaging data analysis (Figure 4b) showed that the percentage of injected dose per gram (%ID/g) of [^18^F]F-TZ(PSMA)-LEGU-TLR7 was significantly higher in PSMA^+^ PC3-PIP (1.9 ± 0.4% ID/g) than in PSMA− PC3-Flu (0.8 ± 0.3% ID/g) tumors (*p* = 0.02). In addition, [^18^F]F-TZ(PSMA)-LEGU-TLR7 was observed with less uptake in the kidneys (3.96 ± 2.1% ID/g) than our previously reported first generation T-SMDC, ^68^Ga-NO3A-DM1-Lys-Urea-Glu (7.33 ± 2.25% ID/g) [29], but with higher accumulation in the heart (6.0 ± 2.2% ID/g), lung (4.7 ± 1.6% ID/g), and liver (25.9 ± 4.6% ID/g). This likely reflects the more lipophilic nature of [^18^F]F-TZ(PSMA)-LEGU-TLR7, which can be attributed to the presence of lipophilic GARD and other aromatic functionalities in the T-SMPDCs (Scheme 1).

## 3. Discussion

The currently broadly-utilized immunotherapy for cancer with immune checkpoint inhibitors relies on a pre-existing immune response. Non-inflamed, immunologically cold tumors are typically resistant to this treatment approach and the primary reason for treatment failure. The capability of inducing de novo immune responses and enhancing otherwise modest benefits is therefore an unmet clinical need in the field of cancer immunotherapy and subject to significant interest and drug development efforts. Although numerous agonists of the TLR pathways have been designed, developed, and tested clinically in past decades [43], their clinical development has been hampered by systemic side effects when delivered systemically or by the need for serial intratumoral injections. TLR7, however, remains an attractive therapeutic target, since it is virtually always present in the microenvironment of solid tumors. TLR7 agonists delivered into the tumor microenvironment can then further diffuse into bystander cells, thus causing a proinflammatory field effect. The antibody–drug conjugate (ADC) strategy has been well-explored for targeted delivery of drug payloads to reduce systemic toxicities [44,45,46]. However, due to the inherent large molecular weight and low tumor-cell penetration ability of antibodies, ADCs are not an optimal choice to deliver TLR agonists to their cytoplasmic targets.

To overcome those limitations, in this work we present a T-SMPDC platform as a feasible solution [29,47]. The prodrug conjugate was designed to reduce the systemic exposure by covalent linkages (to prevent premature drug release) and targeted delivery of the drug payload by a small-molecule platform that can effectively penetrate into tumor microenvironments [29,48]. Additionally, the T-SMPDC platform builds on the mechanism of tumor-specific receptor/antigen-mediated cell internalization to enable intracellular delivery and an enzyme-cleavable prodrug linker to realize the desired controlled release of the drug onto its action target. For the proof-of-concept study, we constructed a theranostic T-SMPDC system, which consists of a model vector, Lys-Urea-Glu, to target cancer-specific PSMA [49], which in tandem initiates cell internalization upon the vector binding [50,51]. The internalized T-SMPDC would then release its drug payload, GARD, via legumain-catalyzed self-immolation of the prodrug linkage in a traceless fashion [33].

For the practicality of T-SMPDC synthesis, we started from 2,4,6-trichloro-1,3,5-triazine and obtained the key intermediate AMP-TZ(Lys-Urea-Glu)-PEG_6_-LEGU-GARD after five steps of synthesis with the overall yield of ~2.3%. Of note, the yield from each of the steps can be further improved by modifying the linker reactivity. The key intermediate, AMP-TZ(Lys-Urea-Glu)-PEG_6_-LEGU-GARD, is stable and can be long-stored for the construction of various T-SMPDCs as necessary. In addition, from the chemistry perspective, it is feasible to scale up the synthesis of AMP-TZ(Lys-Urea-Glu)-PEG_6_-LEGU-GARD. Further step-wise addition of the prodrug moiety, azido-PEG_4_-LEGU-GARD, and the installation of the theranostic [^18/19^F]SFB functionality, were proven straightforward to afford the chemically identical product pair, [^18/19^F]F-TZ(PSMA)-LEGU-TLR7, with reasonable yields. [^18/19^F]F-TZ(PSMA)-LEGU-TLR7 showed the anticipated properties (e.g., serum stability, PSMA binding affinity, PSMA-mediated internalization, and legumain-mediated drug release) and biological behavior (e.g., PSMA-specific uptake and retention in PSMA^+^ tumors), which validate the design concept of our T-SMPDCs for targeted delivery and controlled release of GARD for immunomodulatory therapies. However, we acknowledge that a more clinically relevant animal model must be developed and used in further in vivo theranostic evaluation of the conjugate for immunomodulatory therapies, because TLR7 is expressed in immune cells but our proof-of-concept in vivo studies presented herein were conducted in NOD.CB17-Prkdc^scid^/NCrHsd mice, which lack mature T and B lymphocytes. Therefore, a syngeneic mouse model that retains intact immune systems and carries PSMA positive tumor grafts will be needed to evaluate the immunotherapy potential of our T-SMPDCs. In addition, the in vivo stability of our T-SMPDCs still needs to be improved. A detailed metabolite assay will have to be performed with [^19^F]F-TZ(PSMA)-LEGU-TLR7 in the blood and liver in order to identify the metabolite fragments. Once the chemical bonds are identified as being labile in vivo, we will take corresponding chemical strategies to optimize the structure of the T-SMPDC platform.

Without incorporating an albumin-binding moiety [52], the relatively slow in vivo clearance and tissue-distribution profiles of the T-SMPDCs could be advantageous for the therapeutic applications of the prodrug conjugates because the prolonged high plasma concentration facilitates the targeted accumulation and unidirectional internalization to reach the intended intracellular target protein.

While the in vivo imaging evaluation revealed a relatively high tumor-to-muscle ratio (~4.7) for [^18^F]F-TZ(PSMA)-LEGU-TLR7, it also showed high uptake levels in the heart, lung, and liver. The off-target accumulation likely reflects the lipophilic nature of [^18^F]F-TZ(PSMA)-LEGU-TLR7, which is due to the presence of aromatic functionalities in the structure of T-SMPDCs. Linkers/spacers in the T-SMPDCs can be readily leveraged to overcome the issue [53]. It is noteworthy that the high off-target accumulation of T-SMPDCs is expected to be non-toxic or at least less toxic than the free molecule of GARD, because GARD is covalently loaded to the conjugate. However, whether premature release of GARD would occur or not in the organs need to be investigated. Notably, the bone uptake of [^18^F]F-TZ(PSMA)-LEGU-TLR7 was low (0.79 ± 0.07% ID/g), indicative of the desired in vivo stability of the theranostic moiety. 

The chemical platform of [^18/19^F]F-TZ(PSMA)-LEGU-TLR7 is versatile. The modular synthesis we present in this work can be readily adapted for other targeted therapy systems for cancer precision medicine.

## 4. Materials and Methods

### 4.1. General Materials and Procedures

All chemical reagents and solvents were purchased from commercial sources (Sigma-Aldrich, St. Louis, MO, USA; BroadPharm, San Diego, CA, USA; Fisher Scientific, Hampton, NH, USA) and used as received unless otherwise stated. For aqueous buffer solution preparation, Milli-Q water was obtained from a Millipore Gradient Milli-Q water system (Burlington, MA, USA). Nuclear magnetic resonance (NMR) spectra were recorded on a Bruker 400 MHz NMR (Billerica, MA, USA). Liquid Chromatography-Mass Spectrometry (LC-MS) of compounds were performed by an Agilent 6540 Accurate-Mass Quadrupole Time-of-Flight LC/MS system equipped with 1290 UPLC (Santa Clara, CA, USA). HPLC purifications were performed in an Agilent 1260 Infinity Preparative HPLC system equipped with 1260 photodiode array detector (PDA) and an Agilent Prep-C18 column (150 × 21.2 mm, 5 μm) (Santa Clara, CA, USA). The radiolabeled compounds were characterized by a Waters 600 HPLC system equipped with a Waters 2996 PDA (Milford, MA, USA) and an in-line Shell Jr. 2000 radio detector (Spotsylvania, VA, USA).

### 4.2. Chemistry

#### 4.2.1. Synthesis of TZ-PEG_2_-PROP

A solution of 2,4,6-trichloro-1,3,5-triazine (92 mg, 0.5 mmol) was prepared in 15 mL of anhydrous tetrahydrofuran (THF) under nitrogen (N_2_). *N*,*N’*−diisopropylethylamine (DIPEA) (96 μL, 0.55 mmol) was added to this solution at 0 °C and stirred for 10 min. A solution of propargyl-PEG_2_-amine (71 mg, 0.5 mmol) in THF (4 mL) was added dropwise to this solution at 0 °C and stirring was continued for 3 h. The reaction mixture was then concentrated under vacuum and purified by a reverse-phase HPLC (20% acetonitrile/80% H_2_O to 80% acetonitrile/20% H_2_O over 18 min; all solvents contained 0.1% trifluoroacetic acid (TFA)). The pure compound was obtained by lyophilizing HPLC fractions of TZ-PEG_2_-PROP (52 mg, 36%). MS (ESI) *m*/*z* calcd for C_10_H_12_Cl_2_N_4_O_2_: 290.03; found: 291.03 ([M + H]^+^), Figure S9. ^1^H NMR (400 MHz, CDCl_3_): *δ* 6.48 (s, 1H), 4.21 (s, 2H), 3.77–3.57 (m, 8H), 2.46 (S, 1H), Figure S10. ^13^C NMR (100 MHz, CDCl_3_): *δ* 171.0, 170.1, 165.9, 79.4, 75.0, 70.5, 69.1, 69.1, 58.6, 41.4, Figure S11. Purity of the compound (>95%) was assessed by reverse-phase analytical HPLC.

#### 4.2.2. Synthesis of TZ(PEG_3_-Lys-Urea-Glu)-PEG_2_-PROP (t-Butyl Protected)

TZ-PEG_2_-PROP (43 mg, 0.15 mmol) was dissolved in 5 mL dichloromethane (DCM) under N_2_, to which was added 31 μL of DIPEA (0.18 mmol) at 0 °C. The solution was stirred for 5 min. To this solution, NH_2_-PEG_3_-Lys-Urea-Glu (t-butyl protected) (110 mg, in 5 mL DCM) was gently added and stirred for 12 h at 0 °C. The reaction solvent was evaporated under vacuum in a rotary evaporator to afford a crude product and purified by reverse-phase HPLC (20% acetonitrile/80% H_2_O to 80% acetonitrile/20% H_2_O over 20 min; all solvents contained 0.1% TFA). HPLC fractions were combined from multiple single injections and lyophilized to produce colorless sticky TZ(PEG_3_-Lys-Urea-Glu)-PEG_2_-PROP (t-butyl protected) (68 mg, 46%). MS (ESI) *m*/*z* calcd for C_45_H_78_ClN_9_O_13_: 987.54; found: 988.55 ([M + H]^+^, 494.77 [M/2 + H]^+^), Figure S12. ^1^H NMR (400 MHz, CDCl_3_): *δ* 9.33 (s, 1H), 8.54 (s, 1H), 6.29 (br. s, 2H), 5.88 (br. s, 2H), 4.30–4.18 (m, 4H), 3.75–3.52 (m, 22H), 3.30–3.11 (m, 6H), 2.46 (s, 1H) 2.36–2.28 (m, 2H), 1.94–1.83 (m, 4H), 1.81–1.70 (m, 4H), 1.54–1.32 (m, 27H), 1.30–1.24 (m, 2H), Figure S13. Purity of the compound (>98%) was assessed by reverse-phase analytical HPLC. 

#### 4.2.3. Synthesis of Protected AMP-TZ(PEG_3_-Lys-Urea-Glu)-PEG_2_-PROP

In a 10 mL single-neck round-bottom flask, TZ(PEG_3_-Lys-Urea-Glu)-PEG_2_-PROP (t-butyl protected) (30 mg, 0.03 mmol) was dissolved in DCM/DMF (5 mL/1 mL) under N_2_, to which was added 48 μL of DIPEA (0.29 mmol) under nitrogen at room temperature and stirred for 5 min. At 0 °C, a solution of 4-(aminomethyl)piperidine (AMP) (12 mg, 0.10 mmol) in 5 mL DCM was added dropwise to this solution, which was stirred for 12 h. The reaction solvent was evaporated under vacuum in a rotary evaporator to afford a crude product which was then purified by a reverse-phase HPLC (20% acetonitrile/80% H_2_O to 80% acetonitrile/20% H_2_O over 20 min, all solvents contained 0.1% TFA). HPLC fractions were combined from multiple single injections and lyophilized to produce pure compound as a colorless sticky gel (20 mg, 62%). MS (ESI) *m*/*z* calcd for C_51_H_91_N_11_O_13_: 1065.68; found: 1066.69 ([M + H]^+^, 533.84 [M/2 + H]^+^), Figure S14. Purity of the compound (>99%) was assessed by reverse-phase analytical HPLC. 

#### 4.2.4. Synthesis of AMP-TZ(PEG_3_-Lys-Urea-Glu)-PEG_2_-PROP

Protected AMP-TZ(PEG_3_-Lys-Urea-Glu)-PEG_2_-PROP (20 mg, 0.018 mmol) was dissolved in DCM (1 mL), followed by the addition of TFA (1.5 mL) under N_2_. The reaction mixture was stirred for 12 h at room temperature and the product formation was monitored via ESI-MS. Then, the reaction solvent was evaporated under reduced pressure in a rotary evaporator to afford a crude product, which was then purified by a reverse-phase HPLC (10% acetonitrile/90% H_2_O to 50% acetonitrile/50% H_2_O over 12 min; all solvents contained 0.1% TFA). Fractions from HPLC were combined from multiple single injections and lyophilized to yield pure compound as a sticky liquid (10 mg, 61%). MS (ESI) *m*/*z* calcd for C_39_H_67_N_11_O_13_: 897.49; found: 898.50 ([M + H]^+^, 449.75 [M/2 + H]^+^), Figure S15. ^1^H NMR (400 MHz, CD_3_OD): *δ* 4.33–4.28 (m, 1H), 4.27–4.21 (m, 1H), 4.20–4.14 (m, 2H), 3.69–3.49 (m, 23H), 3.19 (t, 2H), 3.14–3.08 (m, 2H), 3.05–2.99 (m, 2H), 2.91–2.81 (m, 3H), 2.46–2.36 (m, 2H), 2.19–2.10 (m, 1H), 2.03–1.95 (m, 1H), 1.92–1.84 (m, 6H), 1.75–1.69 (m, 2H), 1.53–1.39 (m, 4H), 1.34–1.25 (m, 6H), Figure S16. Purity of the compound (>95%) was assessed by reverse-phase analytical HPLC.

#### 4.2.5. Synthesis of Azido-PEG_4_-LEGU-GARD

Gardiquimod (GARD) (10 mg, 0.03 mmol) was dissolved in THF/DMF (4 mL/2 mL) and stirred for 5 min under N_2_, to which 50 μL of DIPEA (0.3 mmol) was added. The solution was kept stirring for another 15 min under N_2_ at 0 °C. Azido-PEG_4_-Ala-Ala-Asn(Trt)-PAB-PNP (26 mg, 0.025 mmol in THF/DMF (4 mL/2 mL) was added dropwise to this solution at 0 °C and stirred for 24 h. The reaction mixture was then concentrated under vacuum and purified by a reverse-phase HPLC (20% Acetonitrile/80% H_2_O to 90% Acetonitrile/10% H_2_O in 25 min; all solvents contained 0.1 percent TFA). Pure fractions from HPLC were combined from multiple single injections and lyophilized to give a white solid azido-PEG_4_-LEGU-GARD (14 mg, 45%). MS (ESI) *m*/*z* calcd for C_65_H_79_N_13_O_12_: 1233.60; found: 1234.63 ([M + H]^+^), Figure S17. ^1^H NMR (400 MHz, CDCl_3_): *δ* 10.02 (br. s, 1H) 9.14–8.75 (m, 1H), 8.14–7.92 (m, 2H), 7.88–7.39 (m, 7H), 7.19–7.10 (m, 14H), 5.12 (s, 2H), 4.88–4.59 (m, 8H), 4.31–4.16 (m, 2H), 3.68–3.36 (m, 18H), 3.09–2.85 (m, 2H), 2.60–2.31 (m, 2H), 1.45–1.03 (m, 17H), Figure S18. Purity of the compound (>95%) was assessed by reverse-phase analytical HPLC.

#### 4.2.6. Synthesis of AMP-TZ(Lys-Urea-Glu)-PEG_6_-LEGU-GARD

AMP-TZ(PEG_3_-Lys-Urea-Glu)-PEG_2_-PROP (8 mg, 0.009 mmol) and Azido-PEG_4_-LEGU-GARD (11 mg, 0.009 mmol) were dissolved in 1 mL THF at room temperature under N_2_. Copper (II) sulfate pentahydrate (5 mg 0.02 mmol) and sodium ascorbate 4 mg (0.02 mmol) in 0.5 mL of water were charged in a separate reaction vial. The aqueous solution was then added to the organic reaction mixture at room temperature and stirred for 4 hr. The reaction solvent was evaporated under vacuum in a rotary evaporator to afford a crude product which was then purified by a reverse-phase HPLC (20% acetonitrile/80% H_2_O to 80% acetonitrile/20% H_2_O over 25 min; all solvents contained 0.1% TFA). Fractions from HPLC were combined from multiple single injections and lyophilized to produce AMP-TZ(Lys-Urea-Glu)-PEG_6_-LEGU-GARD as a white solid (7 mg, 36%). MS (ESI) *m*/*z* calcd for C_104_H_146_N_24_O_25_: 2131.09; found: 2132.10 ([M + H]^+^, 1066.55 [M/2 + H]^+^, 711.37 [M/3 + H]^+^), Figure S19. ^1^H NMR (400 MHz, CD_3_OD): *δ* 8.48–8.32 (m, 2H), 8.08–7.95 (br. s, 2H), 7.81–7.46 (m, 7H), 7.31–6.99 (m, 12H), 6.95–6.84 (br. s, 2H), 5.40–5.28 (m, 2H), 5.22–5.06 (m, 3H), 4.79–4.70 (m, 3H), 4.67–4.45 (m, 6H), 4.34–4.15 (m, 5H), 3.91–3.82 (m, 2H), 3.75–3.35 (m, 23H), 3.26–2.80 (m, 14H), 2.55–2.32 (m, 6H), 2.23–2.11 (m, 3H), 2.08–1.76 (m, 9H), 1.75–1.53 (m, 6H), 1.52–1.04 (m, 26H), 0.98–0.72 (m, 7H), Figure S20. Purity of the compound (>98%) was assessed by reverse-phase analytical HPLC. 

#### 4.2.7. Synthesis of [^19^F]F-TZ(PSMA)-LEGU-TLR7

AMP-TZ(Lys-Urea-Glu)-PEG_6_-LEGU-GARD (5 mg, 0.0023 mmol) was dissolved in DMF (0.5 mL) under N_2_ at room temperature, to which was added 6 μL of DIPEA (0.03 mmol) at room temperature. The solution was kept stirring for 10 min. N-Succinimidyl 4-fluorobenzoate (0.6 mg 0.0025 mmol) dissolved in 0.2 mL DMF was added to the reaction mixture and stirring was continued for 12 h. The reaction solvent was evaporated to afford a crude product and purified by a reverse-phase HPLC (20% acetonitrile/80% H_2_O to 80% acetonitrile/20% H_2_O over 30 min, all solvents contained 0.1% TFA). HPLC fractions were combined and lyophilized to produce [^19^F]F-TZ(PSMA)-LEGU-TLR7 as a white solid (3 mg, 57%). MS (ESI) *m*/*z* calcd for C_111_H_149_FN_24_O_26_: 2253.11; found: 2254.14 ([M + H]^+^, 1127.56 [M/2 + H]^+^, 752.04 [M/3 + H]^+^), Figure S21. Purity of the compound (>96%) was assessed by reverse-phase analytical HPLC.

### 4.3. Radiochemistry

#### 4.3.1. Production of [^18^F]fluoride

The [^18^F]fluoride production was accomplished on a General Electric PETtrace 880 cyclotron (Chicago, IL, USA). A niobium target body was charged with [^18^O]H_2_O (2.5 mL) for proton bombardment at 60 μA via the ^18^O(p,n)^18^F nuclear reaction to produce [^18^F]fluoride. After 20 min of bombardment, ~55.5 GBq of [^18^F]fluoride was produced.

#### 4.3.2. Azeotropic Drying of [^18^F]fluoride

[^18^F]fluoride (~ 55.5 GBq) was directly transferred to a Synthra RNplus synthesis module and then passed through a QMA Light Sep-Pak cartridge to trap the entire activity. The [^18^F]fluoride was then eluted by a mixture of solution containing aqueous K_2_CO_3_ (1 mg in 0.1 mL H_2_O) and Kryptofix-[2.2.2] (10 mg in 1 mL CH_3_CN) and collected in the reaction vessel (Reactor 1). Then, the reaction mixture was evaporated under vacuum to remove the solvent at 100 °C for 10 min with continuous nitrogen flow.

#### 4.3.3. Synthesis and Purification of [^18^F]N-Succinimidyl 4-Fluorobenzoate, [^18^F]SFB

After complete removal of solvent from reactor 1, trimethylbenzenaminium triflate precursor (5 mg) in 0.5 mL DMSO was added and stirred for 10 min at 125 °C to produce [^18^F]ethyl 4-fluorobenzoate. Tetrapropylammonium hydroxide (TPAH, 1.0 M, 20 μL in 1.0 mL CH_3_CN) was then added to the reaction mixture at 50 °C, followed by a 3 min hydrolysis reaction at 120 °C to produce [^18^F]4-fluorobenzoic acid. The reaction mixture was then evaporated under vacuum at 90 °C with continuous helium flow to remove the solvent. After cooling down the reactor to 50 °C, 10 mg of O-(N-succinimidyl)-N,N,N,N-tetramethyluronium tetrafluoroborate (TSTU in 1 mL CH_3_CN) was added to the reactor 1 and the reaction was continued for 5 min at 90 °C to produce [^18^F]N-succinimidyl 4-fluorobenzoate. The reaction mixture was then transferred to a dilution flask containing 15 mL of 2% acetic acid/98% H_2_O. After dilution, the entire solution mixture was passed through a C18-plus cartridge to trap the crude [^18^F]SFB. The crude [^18^F]SFB was then eluted from the cartridge with 2 mL CH_3_CN and injected into the semi-preparative HPLC. Next, the pure [^18^F]SFB fraction was diluted in 75 mL H_2_O and passed through a C18 plus cartridge to trap the pure [^18^F]SFB. The pure product was then eluted out from the cartridge into reactor 2 with 2.5 mL of CH_3_CN. The solution was evaporated to dryness with helium flow at 48 °C to remove CH_3_CN and water, and then the pure [^18^F]SFB was redissolved in anhydrous CH_3_CN (0.5 mL) for subsequent radiolabeling reaction.

#### 4.3.4. Synthesis of [^18^F]F-TZ(PSMA)-LEGU-TLR7

AMP-TZ(Lys-Urea-Glu)-PEG_6_-LEGU-GARD (0.3 mg), DIPEA (10 μL) and DMSO (100 μL) were mixed at room temperature in a 1.5 mL reaction vial. To this solution, ~ 2.22 GBq of [^18^F]SFB in CH_3_CN (60 μL) was added and incubated for 30 min at 55 °C. The reaction mixture was diluted with 4.2 mL of HPLC mobile phase (39% CH_3_CN/61% H_2_O) and purified with semi-preparative HPLC in the GE TRACERlab FX_FN_ synthesis module. The pure fraction from HPLC containing [^18^F]F-TZ(PSMA)-LEGU-TLR7 was collected in a flask, first mixed with 80 mL of water and then passed through a C18 Plus Sep-Pak cartridge to trap the radiolabeled product. The cartridge was first dried with N2 flow and then eluted with EtOH/H_2_O (90/10; *v*/*v*; 1 mL) to obtain the final product in two-necked flask, which was finally transferred into product vial.

#### 4.3.5. Preparation of ^125^I-Labeled Lys-Urea-Glu Analog

The precursor phloretic acid-PEG_3_-Lys-Urea-Glu (1 mg) was dissolved in 100 μL water to produce a stock solution of 10 μg/μL. A pierce pre-coated iodination tube was rinsed with 1 mL of tris-iodination buffer (5×) and drawn off. Then, 100 μL of tris buffer (5×) was added to the pre-coated tube, followed by 43 MBq of Na^125^I. The iodide was activated for 6 min at room temperature by swirling the tube every 30 s. The activated iodide mixture was then transferred to a reaction vial containing 30 μg of precursor (6) and incubated for 9 min at room temperature. Then, the reaction mixture was purified through a semi-preparative HPLC (5% CH_3_CN/95% H_2_O to 95% CH_3_CN/5% H_2_O over 45 min; all solvents contained 0.1% TFA). Pure fractions from HPLC were diluted in 20 mL of H_2_O and passed through a C18 Plus Sep-Pak cartridge. The final product was eluted from the cartridge with 1 mL ethanol.

### 4.4. Cell Culture and Animal Model

All animal studies were performed in accordance with relevant guidelines and regulations through an animal protocol (APN: 2020-102858; effective from 26 May 2020 to 26 May 2023) approved by the institutional animal care and use committee (IACUC) at the University of Texas Southwestern Medical Center (Dallas, TX, USA). The cell lines, PC3-PIP (PSMA positive) and PC3-Flu (PSMA negative), used in this work have been extensively used in PSMA-targeting agent development [54,55]. They were obtained from the laboratory of Prof. Dr. Martin G. Pomper at John Hopkins University (Baltimore, MD, USA). The sublines of the androgen-independent PC3 human-prostate-cancer cell line, derived from an advanced androgen-independent bone metastasis, were engineered to express a high level of PSMA (PC3-PIP) and maintain no expression of PSMA (PC3-flu) [56,57]. The cells were cultured in RPMI media with the addition of 10% fetal bovine serum, penicillin/streptomycin, and 1 μg/mL of puromycin. For tumor development, cells were cultured at 37 °C under 5% CO_2_ and passaged at 75–90% confluency. Cell suspensions were injected subcutaneously (1.0 × 10^6^ cells in 100 μL Hank’s Buffered Salt Solution) into the thighs of male severe combined immunodeficient (SCID) mice (NOD.CB17-Prkdc^scid^/NCrHsd, 6–8 weeks). The mice were housed in laminar flow cages kept at ~22 °C with ~55% relative humidity in a 12-h light/dark cycle. Throughout the experiment, mice had unrestricted access to autoclaved water and commercial food, and they were checked every other day for general observations and tumor burdens.

### 4.5. Serum Stability Assay

In vitro stability of [^18^F]F-TZ(PSMA)-LEGU-TLR7 was analyzed with human serum. For this purpose, [^18^F]F-TZ(PSMA)-LEGU-TLR7 (1.85 MBq, 20 μL PBS) was added with 400 μL of human serum in a 5 mL quartz glass vial and incubated at 37 °C for 1, 2, and 4 h. Then, 100 μL solution from each was diluted with 1 mL ethanol and centrifuged for 5 min; after the supernatant was filtered out with a 0.2 μm filter, stability was analyzed by radio-HPLC.

### 4.6. Competition Assay

A competitive cell-binding assay of [^19^F]F-TZ(PSMA)-LEGU-TLR7 was performed by utilizing ^125^I-labeled Lys-Urea-Glu as a PSMA targeting radioligand. Suspended PC3-PIP cells were seeded in a multiwell DV plate (Millipore, 5 × 10^4^ cells in 80 μL tris-buffered saline per well). ^125^I-labeled Lys-Urea-Glu (80000 cpm in 20 μL TBS) was then added to each well, followed by the addition of [^19^F]F-TZ(PSMA)-LEGU-TLR7 (3% DMSO in TBS) in increasing concentrations (0, 8, 18, 38, 75, 150, 312, 625, 1250, 2500, 5000, 10,000 nM) and incubated for 2 h (*n* = 4). After incubation, solvents were filtered out and wells were rinsed with cold TBS five times to remove the free radioligand. Radioactivity of each filter was measured by a gamma counter. The best-fit IC_50_ value of [^19^F]F-TZ(PSMA)-LEGU-TLR7 was calculated from the nonlinear regression of the data fitting by using GraphPad Prism 7 (San Diego, CA, USA).

### 4.7. Cell Uptake Assay

Cell-binding potency of [^18^F]F-TZ(PSMA)-LEGU-TLR7 was measured with PSMA^+^ PC3-PIP and PSMA^−^ PC3-Flu cells. Cells were seeded in 6-well plates (5.0 × 10^5^ cells per well, *n* = 3) and incubated for 24 h in a humidified incubator at 37 °C with 5% CO_2_. Afterward, binding buffer (20 mM tris, 150 mM NaCl, pH 7.4) was added to rinse the cells and incubated for 1 h at 37 °C with [^18^F]F-TZ(PSMA)-LEGU-TLR7 (~7.1 × 10^5^ CPM) in 500 μL binding buffer. For the analysis of non-specific binding, PSMA positive and negative cells in 6-well plates were incubated for 1 h with PSMA-targeting ligand Lys-Urea-Glu (1 mM), followed by the addition of [^18^F]F-TZ(PSMA)-LEGU-TLR7 (~7.0 × 10^5^ CPM). Cold binding buffer was added to rinse the cells, three times. Cells were incubated with 0.5 mL 1 M NaOH at 37 °C for 15 min. The NaOH-digested cells were collected in glass tubes to measure the radioactivity counts in a 2480 automatic gamma counter (PerkinElmer, Richmond, CA, USA).

### 4.8. Internalization Assay

Internalization assay was performed with PSMA^+^ PC3-PIP cells. 15-well plates were seeded with 2.0 × 10^5^ cells per well and incubated for 24 h in a humidified incubator at 37 °C with 5% CO_2_. Afterward, a binding buffer (20 mM tris, 150 mM NaCl, pH 7.4) was added to wash the cells. [^18^F]F-TZ(PSMA)-LEGU-TLR7 (~7.2 × 10^5^ CPM) in 400 μL binding buffer was added to each well and incubated for 1, 10, 30, 60, and 120 min (*n* = 6). After each time point, the cells were washed with ice-cold binding buffer. Then, the cells were incubated for 5 min with 0.5 mL ice-cold stripping buffer (150 mM NaCl, 50 mM glycine, pH 3.0) to remove surface-bound [^18^F]F-TZ(PSMA)-LEGU-TLR7. Then, 0.5 mL 1 M NaOH was added and incubated for 15 min at 37 °C to solubilize the cells to collect internalized [^18^F]F-TZ(PSMA)-LEGU-TLR7. The radioactivity counts for both the surface-bound and internalized tracer were counted in a 2480 automatic gamma counter (PerkinElmer).

### 4.9. In Vitro GARD Release Assay by Legumain

Mouse legumain was purchased from Sino Biological Inc. (Chesterbrook, PA, USA) and reconstituted in 25 mM tris-buffered saline (0.15 M NaCl, pH 7.4). GARD release assay was performed in 0.1 M citrate buffer, pH 5.5 (cleavage buffer). 150 μg [^19^F]F-TZ(PSMA)-LEGU-TLR7 was dissolved in 15 μL DMF and diluted to 400 μL with cleavage buffer followed by the addition of 300 μg cysteine. Legumain (7 μg from stock solution) was added to the mixture and incubated at 37 °C. The first aliquot for HPLC and LC/MS was taken after 1 h. The remainder of the sample was set at 37 °C and analyzed by HPLC and LC/MS at different time intervals. A control GARD-release assay was performed under the same condition without legumain protein.

### 4.10. Small Animal PET/CT Imaging

[^18^F]F-TZ(PSMA)-LEGU-TLR7 (ca. 2.9 MBq, in 100 μL PBS) was intravenously injected to tumor-bearing SCID mice (*n* = 3) for small animal PET/CT imaging. Imaging was executed with a Siemens Inveon PET/CT Multimodality System (Knoxville, TN, USA) and the mouse was sedated with 2% isoflurane anesthesia throughout the scan. Static 15 min PET scans were conducted at 1 hr p.i. followed by 7 min CT image acquisition at 80 kV and 500 μA with a focal spot of 58 μm. All the PET and CT data were reconstructed, and regions of interest (ROIs) were marked as displayed by CT to quantify the tracer uptake as percent injected dose per gram of tissue (%ID/g).

## 5. Conclusions

We have successfully designed and developed a unique class of T-SMPDCs for PSMA-targeted delivery and controlled release of TLR7 agonists for immunomodulatory therapies. Further structural optimizations are required to fully unleash the translational potential of this prodrug conjugate system.

## Supplementary Materials

The following supporting information can be downloaded at: https://www.mdpi.com/article/10.3390/ijms23137160/s1, Synthetic procedure of compounds; ^1^H NMR and ^13^C NMR spectra; MS (ESI).
