# Supplementary material for: Theranostic Small-Molecule Prodrug Conjugates for Targeted Delivery and Controlled Release of Toll-like Receptor 7 Agonists
Source: Int J Mol Sci. 2022 Jun 28;23(13):7160. doi: 10.3390/ijms23137160 (PMC9266369; doi:10.3390/ijms23137160)
Supplement: Supplementary file 1 [file ijms-23-07160-s001.zip › ijms-1783010-supplementary.pdf]

Supplementary Materials

Theranostic Small-Molecule Prodrug Conjugates for Targeted Delivery and Controlled Release of Toll-like Receptor 7 Agonists

Sashi Debnath[1], Guiyang Hao[1], Bing Guan[1], Pawan Thapa[1], Justin Hao[1,2], Hans Hammers[*3,4] and Xiankai Sun[*1,4,5]

[1] Department of Radiology, University of Texas Southwestern Medical Center, Dallas, TX 75390, USA; sashi.debnath@utsouthwestern.edu (S.D.); guanbing27@163.com (B.G.); guiyang.hao@utsouthwestern.edu (G.H.);
[2] Texas Academy of Mathematics and Science, University of North Texas, Denton, TX 76203, USA; justinhao@my.unt.edu
[3] Department of Internal Medicine; University of Texas Southwestern Medical Center, Dallas, TX 75390, USA
[4] Kidney Cancer Program; University of Texas Southwestern Medical Center, Dallas, TX 75390, USA
[5] Advanced Imaging Research Center; University of Texas Southwestern Medical Center, Dallas, TX 75390, USA
* Correspondence: hans.hammers@utsouthwestern.edu (H.H.); xiankai.sun@utsouthwestern.edu (X.S.)

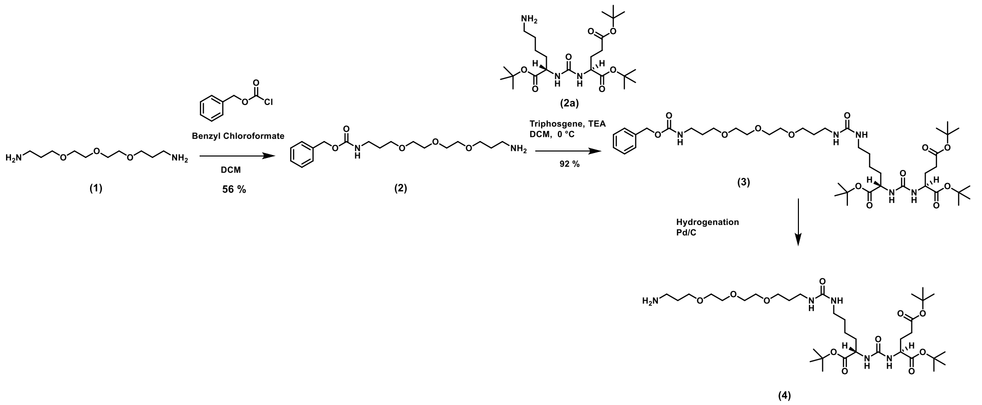

Scheme S1. Synthesis of compound NH_2-PEG_3-Lys-Urea-Glu (t-butyl protected) (4).

Synthesis of compound S2

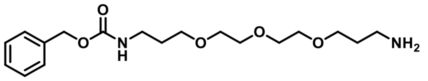

(2)

4,7,10-Trioxa-1,13-tridecanediamin (10.52 mL, 48 mmol) was diluted in 150 mL CH_2Cl_2 under nitrogen at 0 °C. Then the mixture was stirred at 0 °C for 20 minutes, followed by the addition of benzoyl chloroformate (853 μL, 6 mmol) solution in 20 ml THF. Stirring was continued at room temperature for 16 h. CH_2Cl_2 layer was extracted from water and concentrated in a rotary evaporator. Pure compound was isolated as a colorless syrup from silica gel column chromatography, 20 % methanol in CH_2Cl_2 solvent mixture (1.2 g, 56%). ^{1}H NMR (400 MHz, $CDCl_3$): δ 7.32 (m, 5H), 5.61 (s, 2H), 5.07 (s, 2H), 3.57-3.52 (m, 10H), 3.29 (s, 2H), 2.78 (s, 2H), 2.42 (s, 2H), 1.76-1.68 (m, 4H). Figure S1.

Synthesis of compound S3

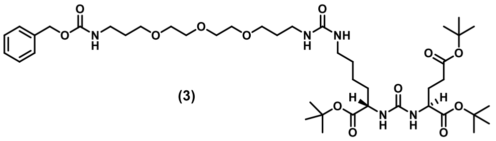

Compound **2** (198 mg, 0.56 mmol) and triphosgene (70 mg, 0.23 mmol) were diluted in CH_2Cl_2 (25 mL) at 0 °C under nitrogen and then Et_3N (150 mg, 1.5 mmol) was added. The mixture was stirred at 0 °C for 2 h to allow isocyanate formation, followed by the addition of compound **2a** (300 mg, 0.61 mmol) in Et_3N (80 mg, 0.8 mmol) and CH_2Cl_2 (3 mL). Stirring was continued at room temperature for 16 h, and then the reaction was quenched with 1 M HCl (30 mL). The organic layer was concentrated into a yellow syrup, which was purified by flash column chromatography, eluting with a 5 % MeOH in CH_2Cl_2 solvent mixture to provide compound **3** as a clear colorless syrup (450 mg, 92%). MS (ESI) *m/z* calcd for $C_{43}H_{73}N_5O_{13}$: 867.52; found: 868.54 ($[M + H]^+$, Figure S2.

Synthesis of NH_2-PEG_3-Lys-Urea-Glu (t-butyl protected), S4

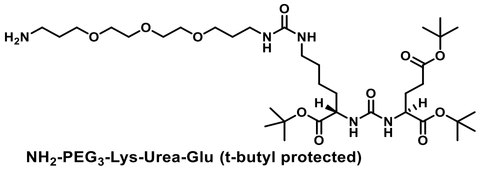

Compound **3** (0.26 g, 0.3 mmol) was diluted in EtOAc (15 mL) and degassed for 5 min with nitrogen, followed by the addition of 10% Pd on activated charcoal (30 mg), and the mixture was degassed for another 5 min. The mixture was hydrogenated at room temperature with a hydrogen balloon for 36 h and was then filtered and washed with EtOAc through a Celite pad. The solution was concentrated to provide a syrup which was purified by flash column chromatography, eluting with a 10% gradient of MeOH in CH_2Cl_2, to give the compound **4** as a clear colorless syrup (0.19 g, 86%). MS (ESI) *m/z* calcd for $C_{35}H_{67}N_5O_{11}$: 733.48; found: 734.51 ($[M + H]^+$, Figure S3, SI. ^1H NMR (400 MHz, $CDCl_3$): δ 8.02-7.77 (m, 4H), 4.31-4.18 (m, 2H), 3.77-3.45 (m, 14H), 3.29-3.10 (m, 6H), 2.39-2.28 (m, 2H), 2.12-1.93 (m, 4H), 1.85-1.66 (m, 4H), 1.58-1.33 (m, 27H), 1.31-1.22 (m, 2H), Figure S4.

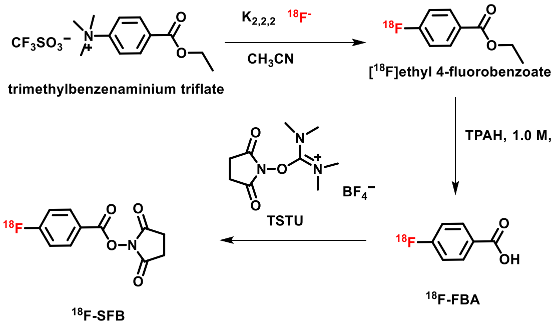

Scheme S2. Synthesis of [^{18}F]N-Succinimidyl 4-fluorobenzoate, [^{18}F]SFB.

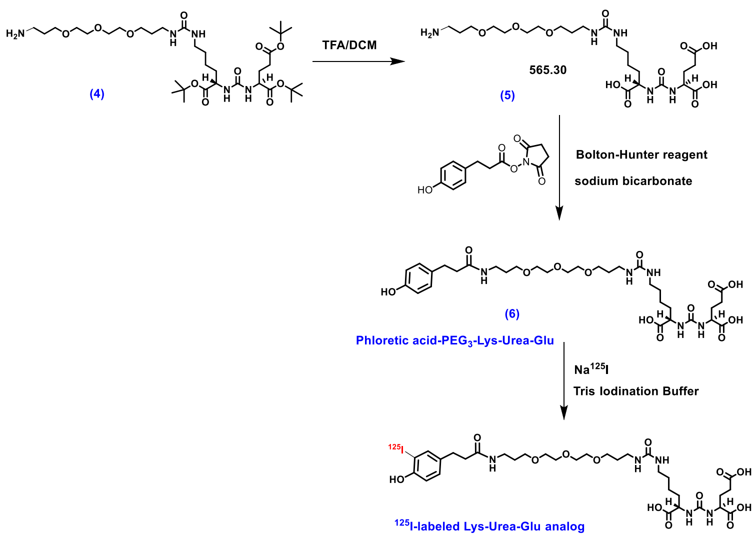

Scheme S3. Synthesis of ^{125}I-labeled Lys-Urea-Glu analog.

Synthesis of compound S5

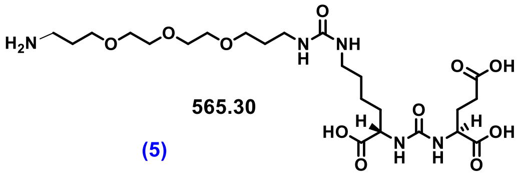

Compound **4** (73 mg, 0.1 mmol) was dissolved in DCM (2 mL), and trifluoroacetic acid (2.0 ml) was added to this solution under nitrogen (N_2). The reaction mixture was then stirred for 6 h, and product formation was monitored via ESI-MS. The reaction solvent was evaporated under reduced pressure in a rotary evaporator to afford a crude product which was then purified by a reversed phase HPLC (10% Acetonitrile/90% H_2O to 40% Acetonitrile/60% H_2O over 14 min, all solvent contained 0.1% TFA). Pure fractions from HPLC were combined from multiple single injections and lyophilized to produce compound **5** as a sticky liquid (39 mg, 70%). MS (ESI) *m/z* calcd for $C_{23}H_{43}N_5O_{11}$: 565.30; found: 566.37 ($[M + H]^+$, Figure S5.

Synthesis of Phloretic acid-PEG3-Lys-Urea-Glu, S6

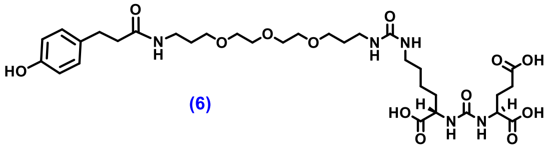

Compound **5** (29 mg, 0.05 mmol) was diluted in a 1:1 mixture of acetone and water (2 mL) under nitrogen, and Bolton-Hunter reagent precursor (16 mg, 0.06 mmol) was added to this solution. Then the mixture was stirred at room temperature for 5 minutes, followed by the addition of $NaHCO_3$ (16.8 mg, 0.20 mmol). Stirring was continued for 12 h at room temperature. The crude reaction mixture was then purified by a reversed phase HPLC (10% Acetonitrile/90% H_2O to 60% Acetonitrile/40% H_2O over 15 min; all solvents contained 0.1% TFA). Pure fractions from HPLC were combined together from multiple single injections and lyophilized to produce compound **6** as a white solid (22 mg, 61%). MS (ESI) *m/z* calcd for $C_{32}H_{51}N_5O_{13}$: 713.35; found: 714.42 ($[M + H]^+$, Figure S6, SI. ^{1}H NMR (400 MHz, D_2O): δ 7.14 (d, J=8.52, 2H), 6.85 (d, J=8.52,

2H), 4.30-4.13 (m, 2H), 3.70-3.47 (m, 10H), 3.24-3.04 (m, 8H), 2.90-2.80 (m, 2H), 2.55-2.45 (m, 4H), 2.23-2.11 (m, 1H), 2.04-1.90 (m, 1H), 1.87-1.64 (m, 4H), 1.61-1.32 (m, 6H), Figure S7, SI. ^{13}C NMR (100 MHz, D_2O): δ 177.22, 175.51, 160.53, 159.23, 155.00, 153.78, 151.10, 132.12, 129.75, 115.27, 69.49, 69.44, 69.24, 69.21, 68.44, 67.95, 53.22, 52.62, 39.48, 37.59, 36.91, 35.90, 30.61, 30.48, 30.01, 29.01, 28.62, 28.01, 26.25, 22.18, Figure S8.

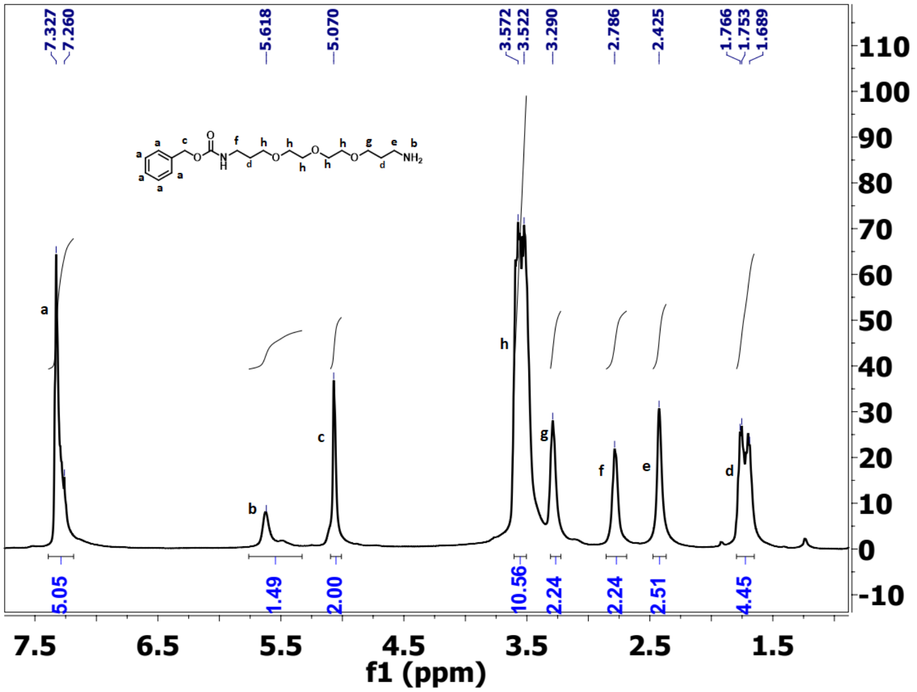

Figure S1 ^{1}H NMR of compound **2**.

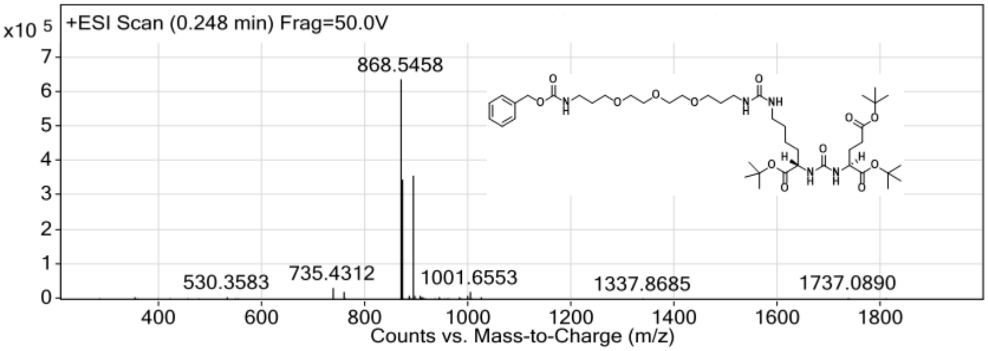

Figure S2 MS (ESI) of compound **3**.

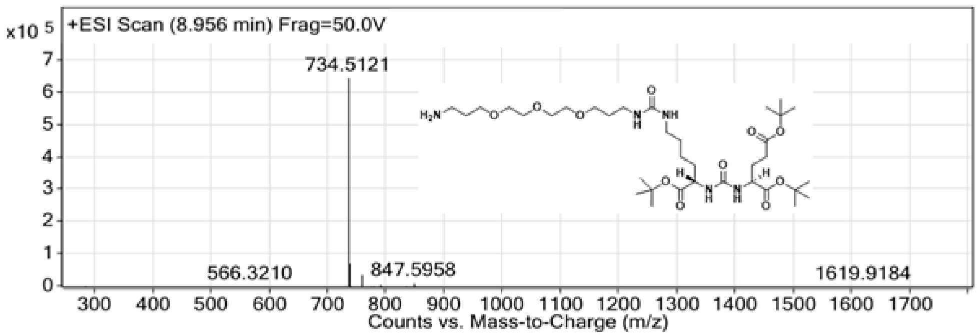

Figure S3 MS (ESI) of compound **4**.

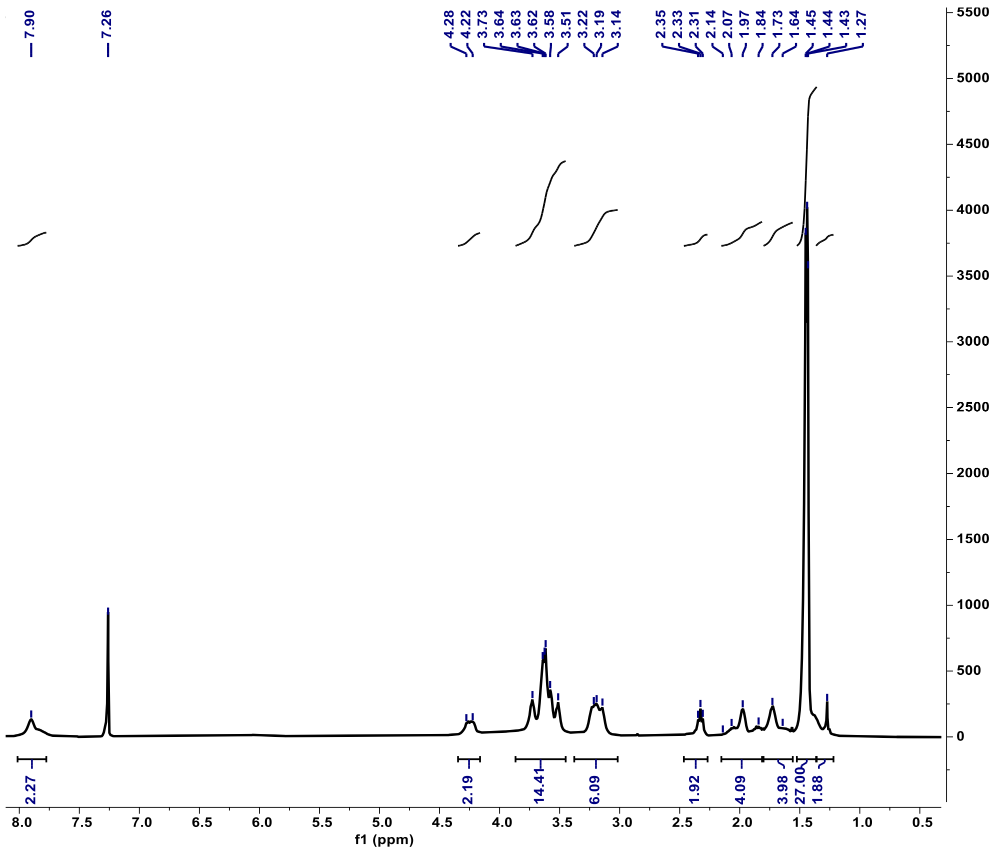

Figure S4 ^{1}H NMR of compound **4**.

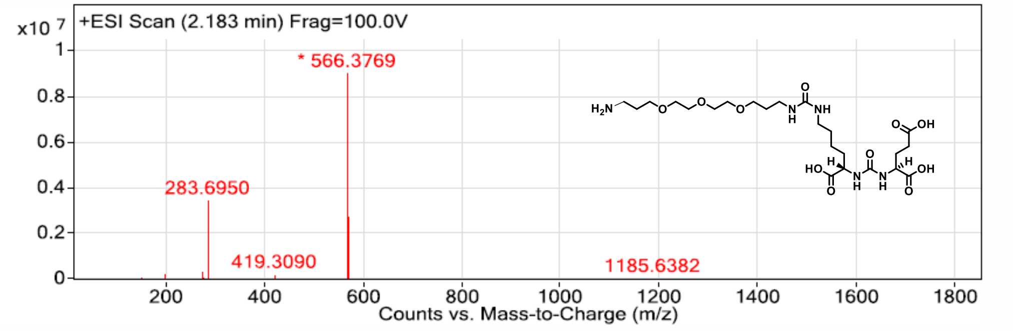

Figure S5 MS (ESI) of compound **5**.

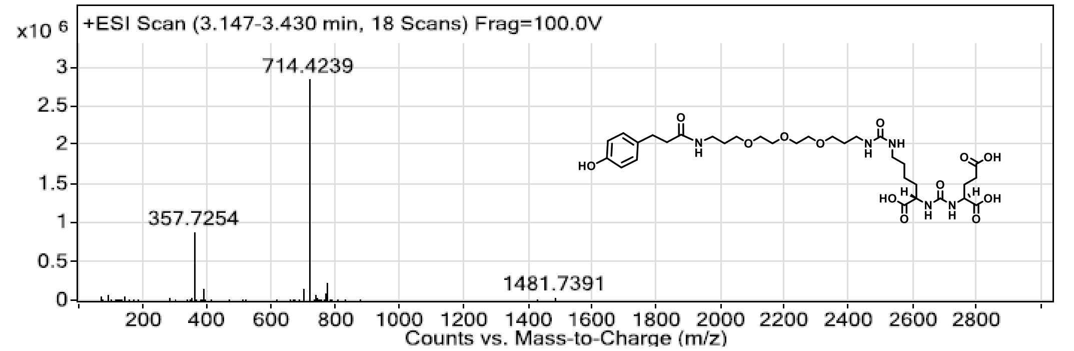

Figure S6 MS (ESI) of compound **6**.

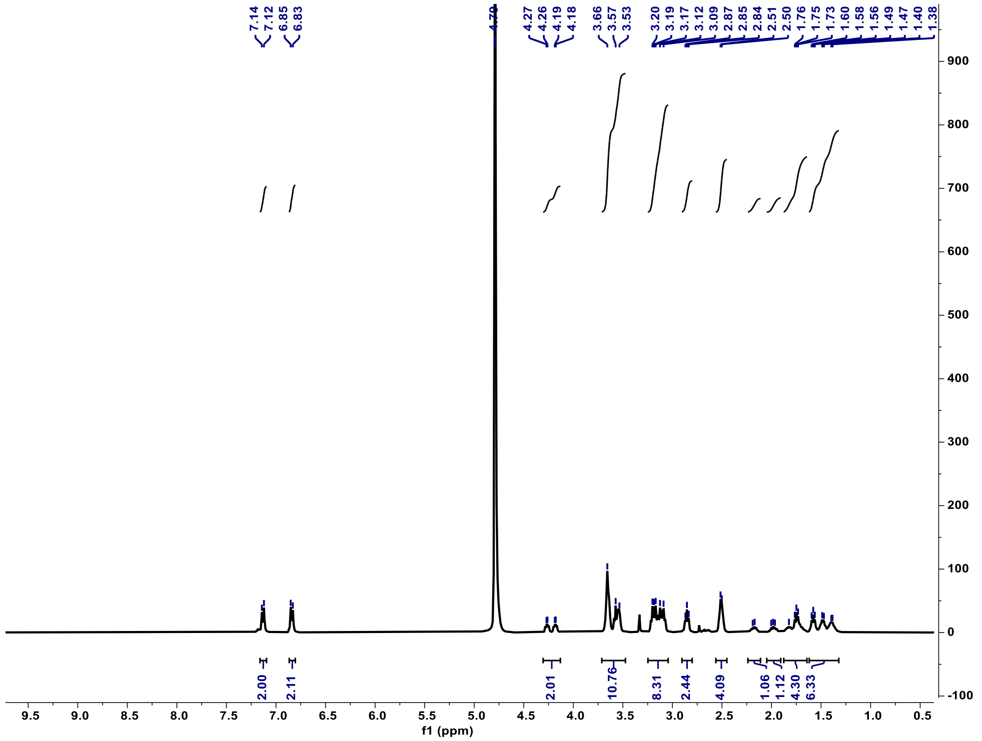

Figure S7 ^{1}H NMR of compound **6**.

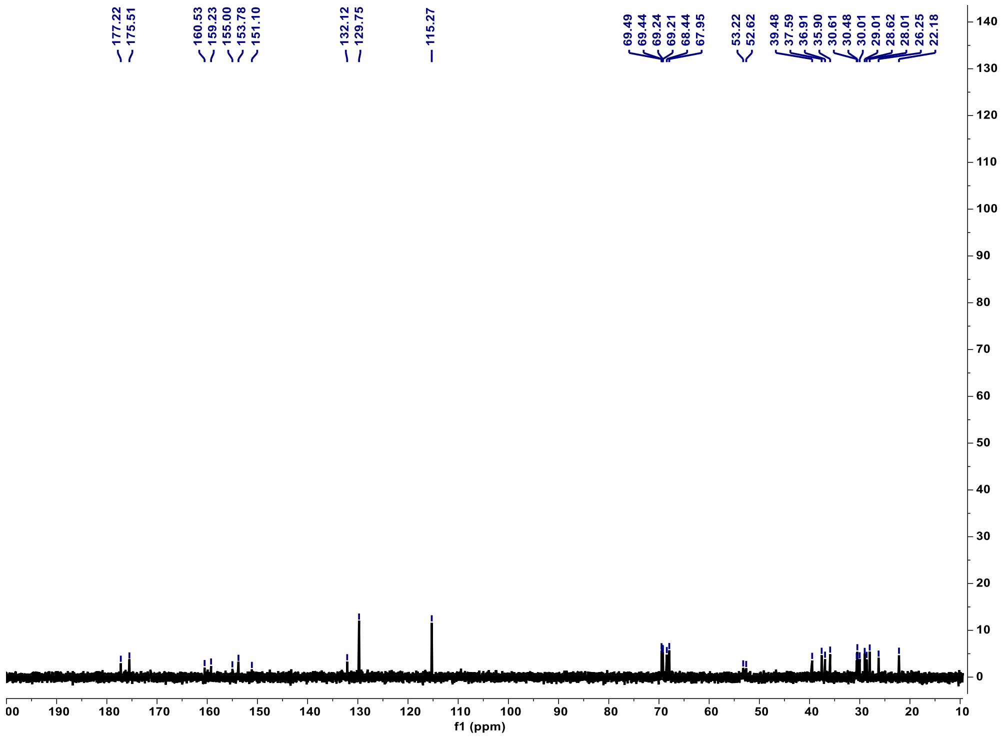

Figure S8 ^{13}C NMR of compound **6**.

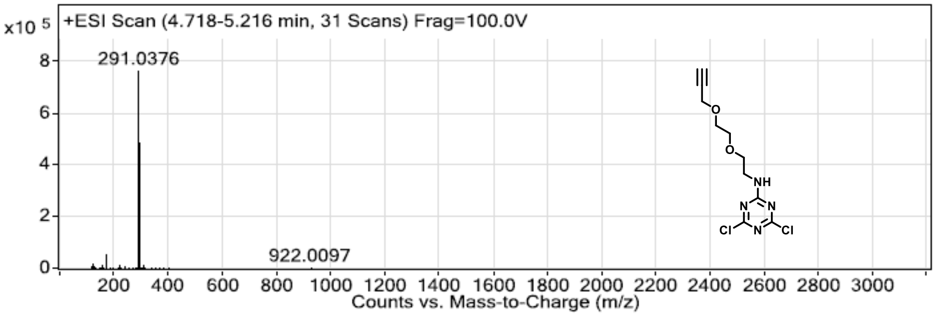

Figure S9 MS (ESI) of compound TZ-PEG$_2$-PROP.

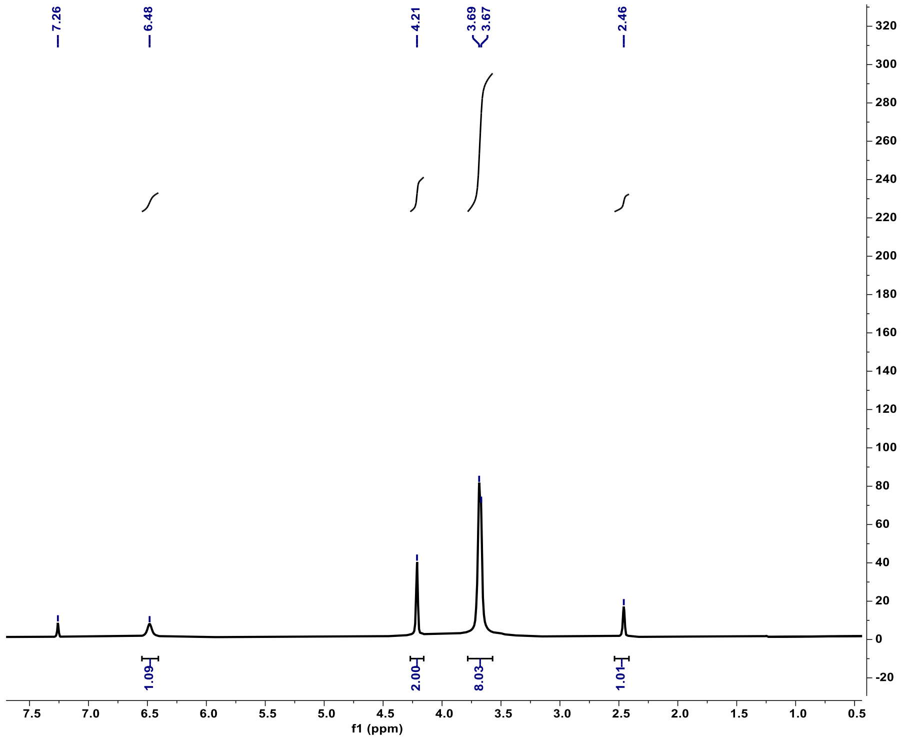

Figure S10 ¹H NMR of compound TZ-PEG₂-PROP.

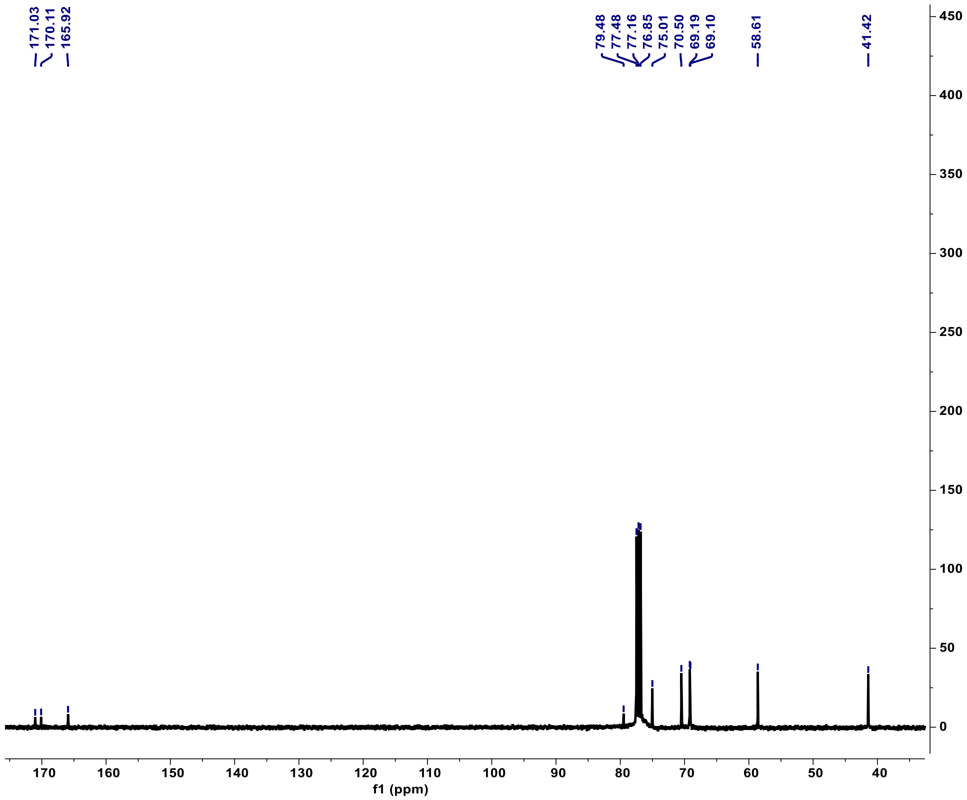

Figure S11 ¹³C NMR of compound TZ-PEG₂-PROP.

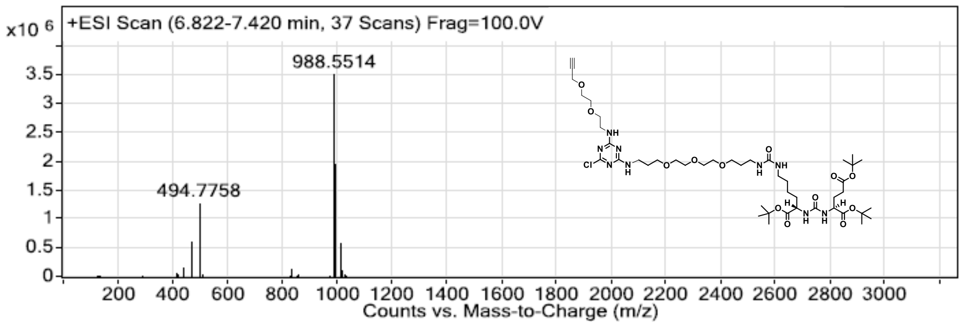

Figure S12 MS (ESI) of compound TZ(PEG3-Lys-Urea-Glu)-PEG2-PROP (t-butyl protected).

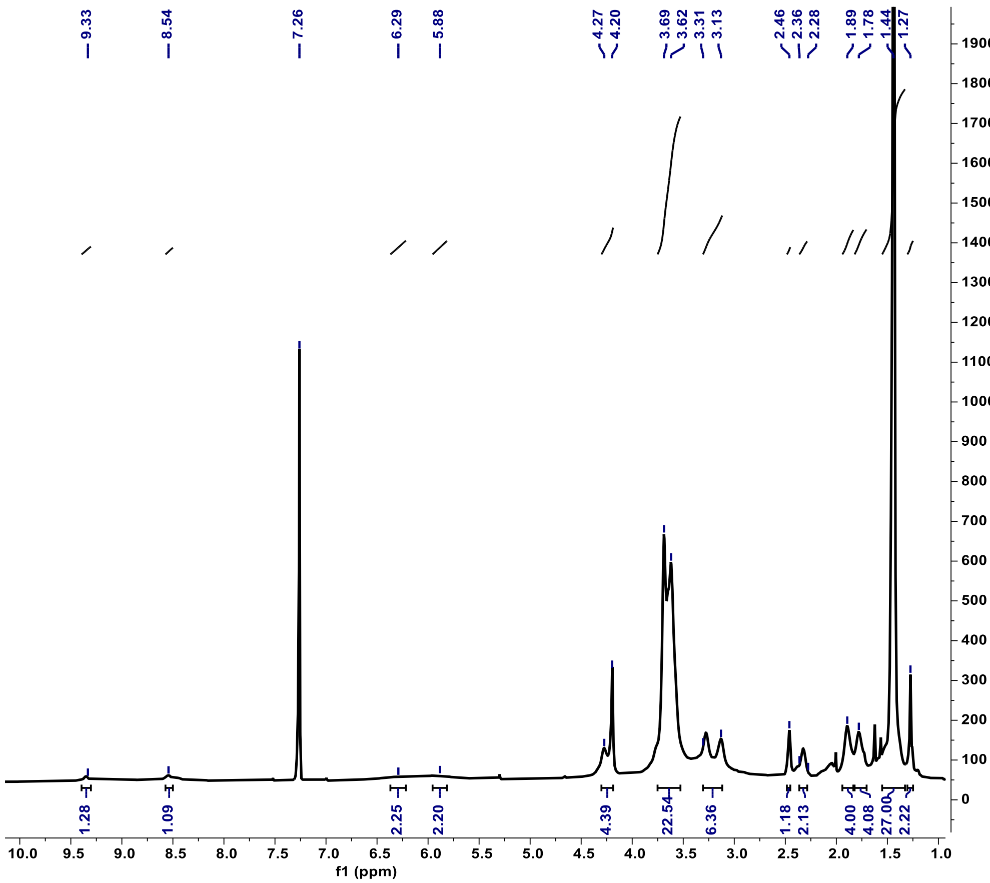

Figure S13 ^{1}H NMR of compound TZ(PEG3-Lys-Urea-Glu)-PEG2-PROP (t-butyl protected).

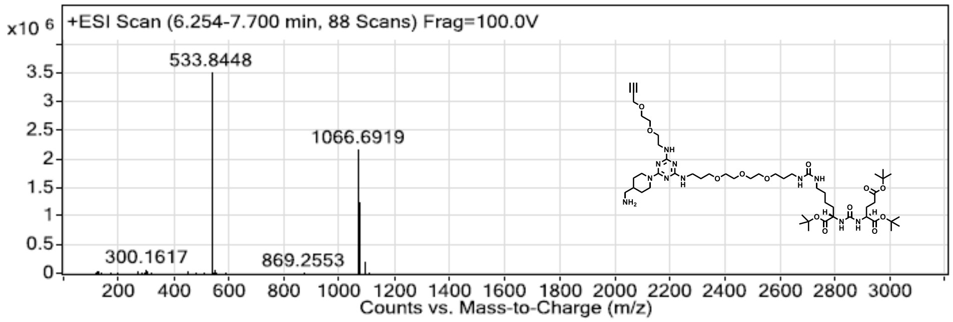

Figure S14 MS (ESI) of compound protected-AMP-TZ(PEG$_3$-Lys-Urea-Glu)-PEG$_2$-PROP.

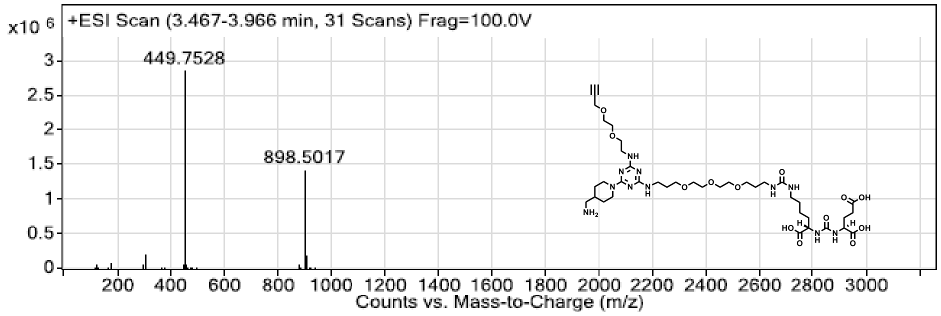

Figure S15 MS (ESI) of compound AMP-TZ(PEG$_3$-Lys-Urea-Glu)-PEG$_2$-PROP.

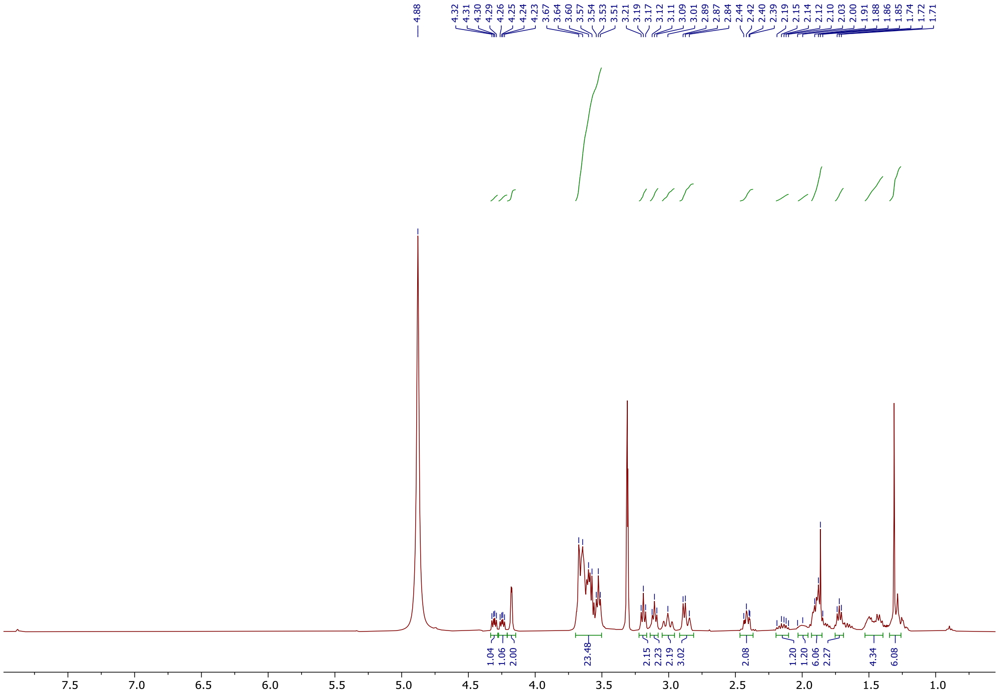

Figure S16 ^{1}H NMR of compound AMP-TZ(PEG$_3$-Lys-Urea-Glu)-PEG$_2$-PROP.

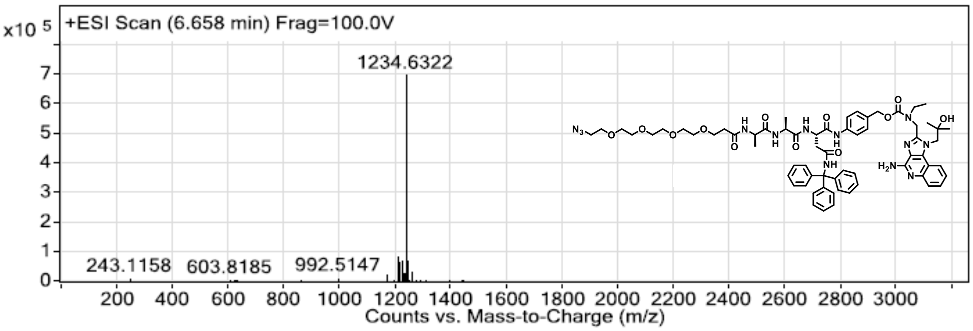

Figure S17 MS (ESI) of compound Azido-PEG$_4$-LEGU-GARD.

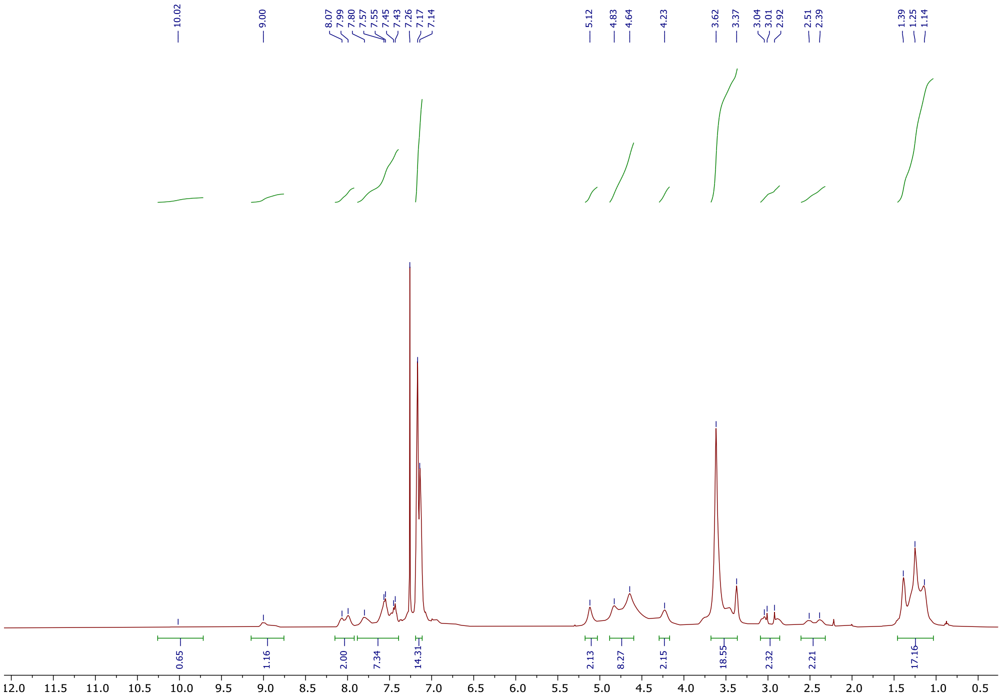

Figure S18 ^{1}H NMR of compound Azido-PEG$_4$-LEGU-GARD.

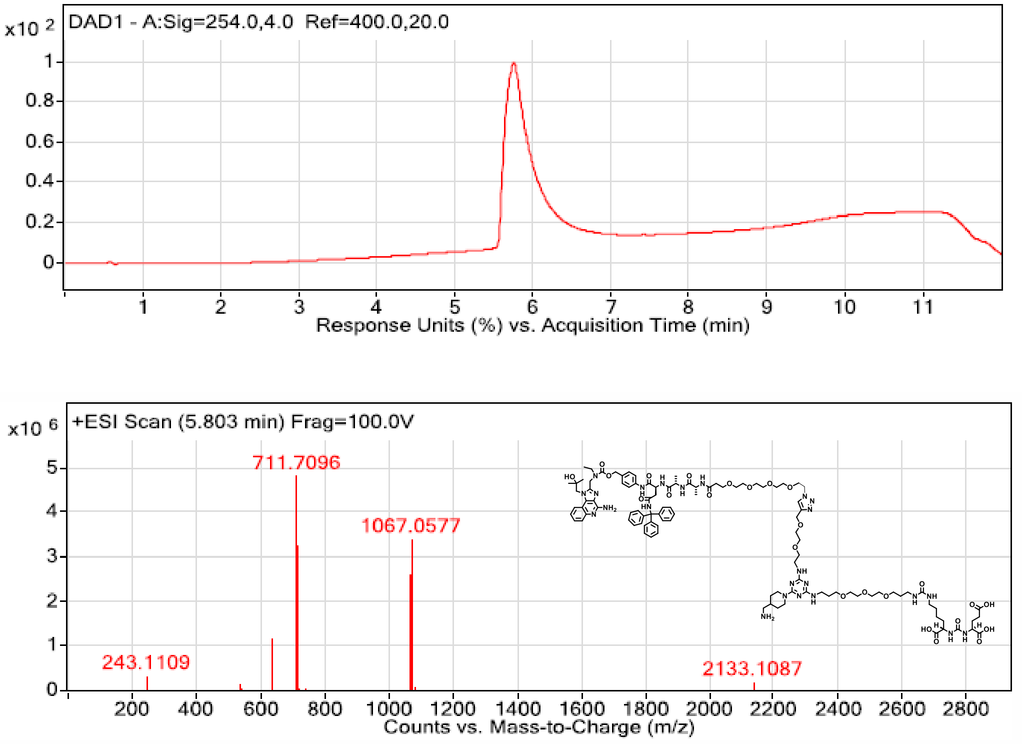

Figure S19 MS (ESI) of compound AMP-TZ(Lys-Urea-Glu)-PEG$_6$-LEGU-GARD.

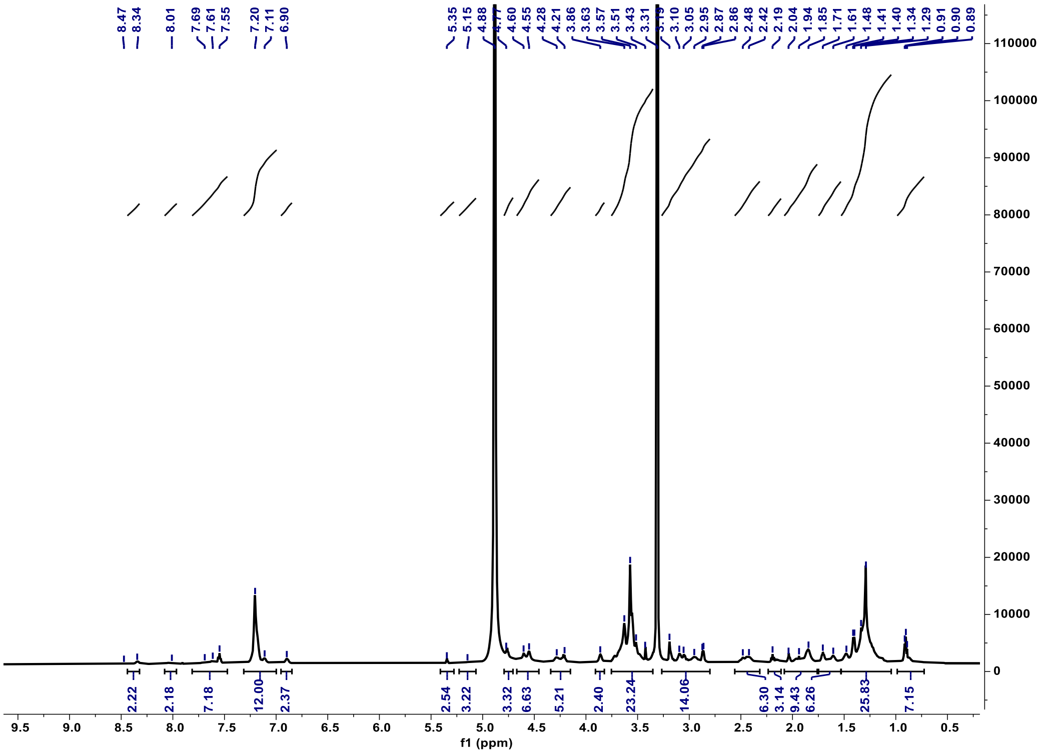

Figure S20 ^{1}H NMR of compound AMP-TZ(Lys-Urea-Glu)-PEG6-LEGU-GARD

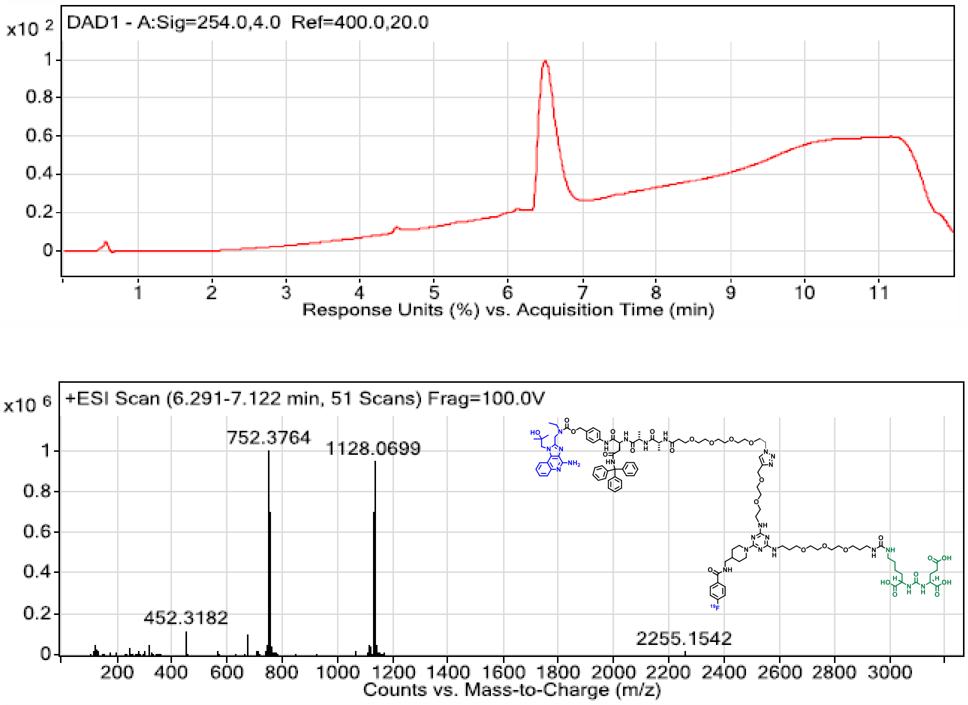

Figure S21 MS (ESI) of compound [19F]F-TZ(PSMA)-LEGU-TLR7.

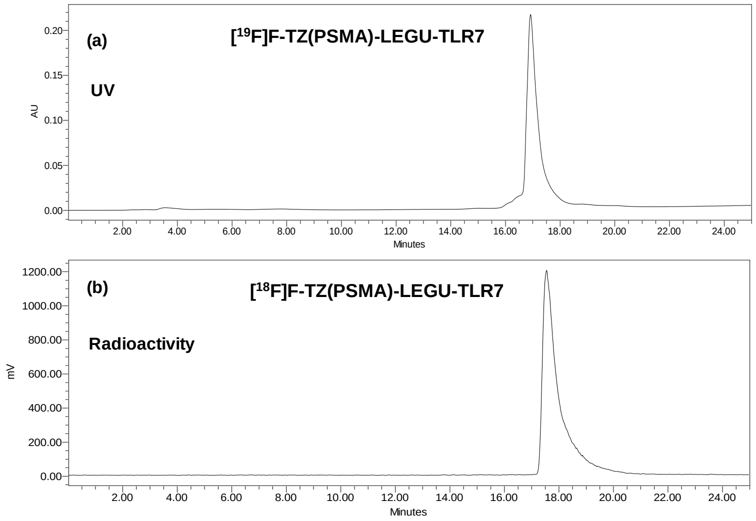

Figure S22. Chemical identity of radiolabeled product [18F]F-TZ(PSMA)-LEGU-TLR7 with standard [19F]F-TZ(PSMA)-LEGU-TLR7. HPLC chromatograms of [19F]F-TZ(PSMA)-LEGU-TLR7 (a) and [18F]F-TZ(PSMA)-LEGU-TLR7 (b).

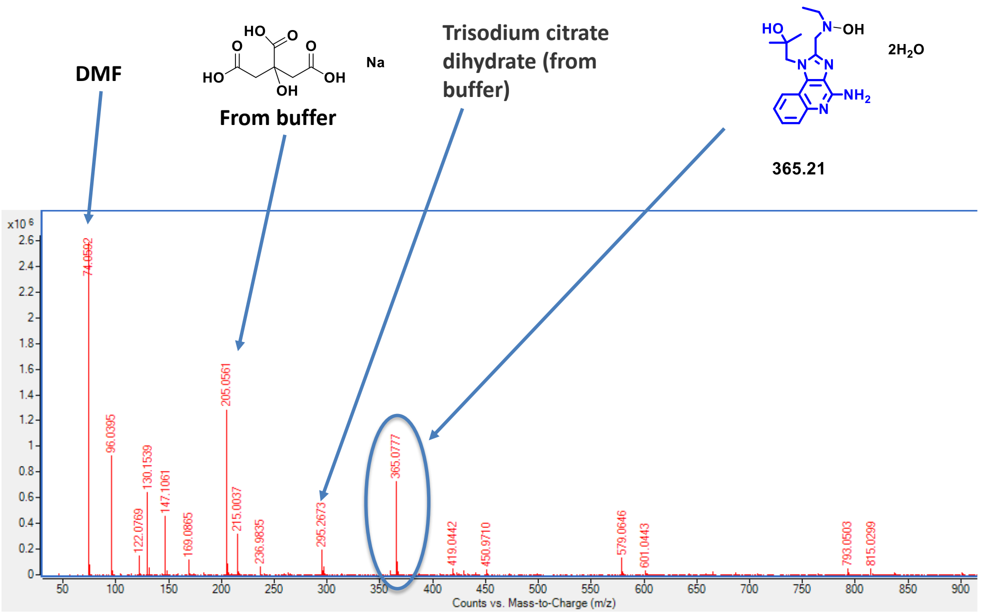

Figure S23. MS (ESI) of legumain treated conjugate [19F]F-TZ(PSMA)-LEGU-TLR7.

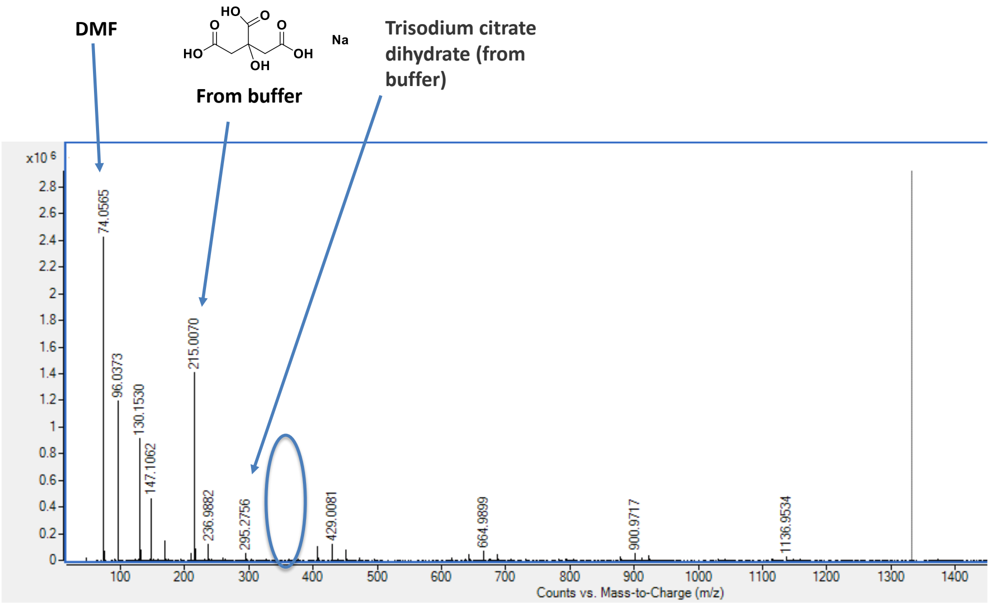

Figure S24. MS (ESI) of legumain untreated conjugate [19F]F-TZ(PSMA)-LEGU-TLR7.
